# Sharp Conditions for the BBM Formula and Asymptotics of Heat Content-Type Energies

**DOI:** 10.1007/s00205-025-02157-1

**Published:** 2026-01-07

**Authors:** Luca Gennaioli, Giorgio Stefani

**Affiliations:** 1 Mathematics Research Centre, University of Warwick, CV4 7AL Coventry, UK; 2 Dipartimento di Matematica “Tullio Levi-Civita”, Università degli Studi di Padova, Via Trieste 63, 35121 Padua, PD Italy

## Abstract

Given $p\in [1,\infty )$, we provide sufficient and necessary conditions on the non-negative measurable kernels $(\rho _t)_{t\in (0,1)}$ ensuring convergence of the associated Bourgain–Brezis–Mironescu (BBM) energies $(\mathscr {F}_{t,p})_{t\in (0,1)}$ to a variant of the *p*-Dirichlet energy on $\mathbb {R}^N$ as $t\rightarrow 0^+$ both in the pointwise and in the $\Gamma$-sense. We also devise sufficient conditions on $(\rho _t)_{t\in (0,1)}$ yielding local compactness in $L^p(\mathbb {R}^N)$ of sequences with bounded BBM energy. Moreover, we give sufficient conditions on $(\rho _t)_{t\in (0,1)}$ implying pointwise and $\Gamma$-convergence and equicoercivity of $({\mathscr {F}}_{t,p})_{t\in (0,1)}$ when the limit *p*-energy is of non-local type. Finally, we apply our results to provide asymptotic formulas in the pointwise and $\Gamma$-sense for heat content-type energies both in the local and non-local settings.

## Introduction

### Framework

We let $I=(0,1)$ and we fix a family $(\rho _t)_{t\in I}\subset L^1_\textrm{loc}(\mathbb {R}^N)$ of non-negative functions. Given $p\in [1,\infty )$ and $t\in I$, we consider the non-local functionals ${\mathscr {F}}_{t,p}:L^p(\mathbb {R}^N)\rightarrow [0,\infty ]$ defined as

1.1 $$\begin{aligned} \begin{aligned} {\mathscr {F}}_{t,p}(u) = \int _{\mathbb {R}^N} \int _{\mathbb {R}^N} \frac{|u(x)-u(y)|^p}{|x-y|^p}\,\rho _t(x-y)\,\textrm{d}x\,\textrm{d}y \end{aligned} \end{aligned}$$

for all $$u\in L^p(\mathbb {R}^N)$$.

The asymptotic behavior of the family $(\mathscr {F}_{t,p})_{t \in I}$ as $t \rightarrow 0^+$ was first investigated by Bourgain, Brezis and Mironescu in their seminal paper [20]. Their work has inspired a vast literature on non-local-to-local convergence results (or *BBM formulas*, after [20]).

While a comprehensive overview of the research is beyond our scope, we focus on the results most aligned with the spirit of [20]. In particular, we refer to [12, 32, 54, 55, 61, 62] for foundational contributions and to [51, 52] for extensions to general open sets. For $\Gamma$-convergence results, see [10, 15, 30, 36, 47, 60]. For energies derived from gradient-type integro-differential operators, we refer to [14, 26, 27, 29, 56, 57, 64]. For the extension to non-Euclidean frameworks, such as magnetic Sobolev spaces, Riemannian manifolds, Carnot groups, and metric-measure spaces, see [13, 37, 41–46, 48, 49, 59]. For other strictly related results we refer to the monographs [6, 53].

BBM formulas play a central role in several applications in modern Analysis. Far from being complete, we refer to Brezis’ celebrated work [23] on how to recognize constant functions, to [25] for applications to image denoising, to [33] for extensions accounting for antisymmetric exchange interactions, and to [7, 65] for the study of the convergence of non-local Ginzburg–Landau functionals.

### Sharp Conditions

A common trait of the works mentioned above is that they only concern *sufficient* conditions for BBM formulas to hold. Namely, in the specific case of the functionals in (1.1), under a certain set of conditions on the family $(\rho _t)_{t\in I}$, one can find an infinitesimal sequence $(t_k)_{k\in \mathbb {N}}\subset I$ and a non-negative Radon measure $\mu \in {\mathscr {M}}^+({\mathbb {S}}^{N-1})$ on the $(N-1)$-dimensional sphere ${\mathbb {S}}^{N-1}$ in $\mathbb {R}^N$, depending on $(\rho _{t_k})_{k\in \mathbb {N}}$ only, such that

1.2 $$\begin{aligned} \lim _{k\rightarrow \infty } {\mathscr {F}}_{t_k,p}(u) = {\mathscr {D}}_p^\mu (u) \end{aligned}$$

for every $u\in {\mathcal {S}^{p}}(\mathbb {R}^N)$, where

1.3 $$\begin{aligned} {\mathscr {D}}_p^\mu (u) = \int _{{\mathbb {S}}^{N-1}}\Vert \sigma \cdot Du\Vert _{L^p}^p\,\textrm{d}\mu (\sigma ). \end{aligned}$$

Here and in what follows, we let

$$\begin{aligned} {\mathcal {S}^{p}}(\mathbb {R}^N) = {\left\{ \begin{array}{ll} W^{1,p}(\mathbb {R}^N) &  \text {for}\ p>1, \\ BV(\mathbb {R}^N) &  \text {for}\ p=1, \end{array}\right. } \end{aligned}$$

and we let *Du* be the distributional gradient of $u\in {\mathcal {S}^{p}}(\mathbb {R}^N)$ (if $p=1$, then *Du* may be a finite Radon measure on $\mathbb {R}^N$). Moreover, for every $\sigma \in {\mathbb {S}}^{N-1}$ and $u\in {\mathcal {S}^{p}}(\mathbb {R}^N)$, we let

$$\begin{aligned} \Vert \sigma \cdot Du\Vert _{L^p}^p = {\left\{ \begin{array}{ll} \displaystyle \int _{\mathbb {R}^N}|\sigma \cdot Du(x)|^p\,\textrm{d}x &  \text {for}\ p>1, \\ |\sigma \cdot Du|(\mathbb {R}^N) &  \text {for}\ p=1. \end{array}\right. } \end{aligned}$$

In the recent paper [34], the authors devise a set of conditions on the family $(\rho _t)_{t\in I}$ that are both *sufficient* and *necessary* for the validity of (1.2) in the case $p=2$ by means of Fourier transform techniques. Precisely, they recast the functionals $({\mathscr {F}}_{t,2})_{t\in I}$ in (1.1) into double integrals of the form

$$\begin{aligned} v\mapsto \int _{\mathbb {R}^N}|v(\xi )|^2\int _{\mathbb {R}^N}\frac{1-\cos (z\cdot \xi )}{|z|^2}\,\rho _t(z)\,\textrm{d}z\,\textrm{d}\xi , \end{aligned}$$

where $v\in L^2(\mathbb {R}^N)$ is such that $|\cdot |\,|v|\in L^2(\mathbb {R}^N)$. Unfortunately, for $p\ne 2$, the Fourier approach is not viable anymore, but in [34, Sect. 5.3] the authors conjecture that similar conditions are sufficient and necessary for the validity of (1.2) for every $p\in [1,\infty )$.

For *radially symmetric* families $(\rho _t)_{t\in I}$, necessary conditions are outlined in [39] for $p=2$, while sufficient and necessary conditions are achieved in [38] for every $p>1$, confirming the conjecture made in [34] in this particular case.

To the best of our knowledge, the conjecture in [34] is currently open for arbitrary families $(\rho _t)_{t\in I}$ and $p\ne 2$. Our first main result, stated in Theorem 1.1, below, affirmatively answers the conjecture posed in [34]. Even more, we prove that the conditions devised in [34] are sufficient and necessary for the convergence of the functionals in (1.1) not only in the *pointwise* sense, but also in the $\Gamma$*-convergence* sense (for a complete description of $\Gamma$*-convergence*, we refer to the monographs [21, 31]). For the notation, we refer to Sect. 2.

#### Theorem 1.1

Let $p\in [1,\infty )$. The following are equivalent:

A There exists an infinitesimal sequence $(t_k)_{k\in \mathbb {N}}\subset I$ such that
  1.4 $$\begin{aligned} \sup _{R>0} \limsup _{k\rightarrow \infty } R^p\int _{\mathbb {R}^N}\frac{\rho _{t_k}(z)}{R^p+|z|^p}\,\textrm{d}z<\infty \end{aligned}$$
  and $\nu _k=\rho _{t_k}{\mathscr {L}}^N\overset{\star }{\rightharpoonup }\alpha \delta _0$ in ${\mathscr {M}}_\textrm{loc}(\mathbb {R}^N)$ as $k\rightarrow \infty$ for some $\alpha \ge 0$.
B There exist an infinitesimal sequence $(t_k)_{k\in \mathbb {N}}\subset I$ and $\mu \in {\mathscr {M}}^+({\mathbb {S}}^{N-1})$ such that:
  i if $$u\in {\mathcal {S}^{p}}(\mathbb {R}^N)$$, then $$\displaystyle \limsup _{k\rightarrow \infty } {\mathscr {F}}_{t_k,p}(u) \le {\mathscr {D}}_p^\mu (u)$$;
  ii if $(u_k)_{k\in \mathbb {N}}\subset L^p(\mathbb {R}^N)$ is such that $u_k\rightarrow u$ in $L^p(\mathbb {R}^N)$ as $k\rightarrow \infty$ for some $u\in {\mathcal {S}^{p}}(\mathbb {R}^N)$, then $\displaystyle \liminf _{k\rightarrow \infty } {\mathscr {F}}_{t_k,p}(u_k) \ge {\mathscr {D}}_p^\mu (u)$.

In parts (A) and (B) of Theorem 1.1, the sequence $(t_k)_{k \in \mathbb {N}}$ is not necessarily the same. We refer to Remark 3.2 for an example where one must pass to a subsequence of $(t_k)_{k\in \mathbb {N}}$ in the implication (A)$\implies$(B). Moreover, as observed in [51, 61], property (ii) in (B) can be further refined by additionally requiring that the family $(\rho _t)_{t\in I}$ has *maximal rank* (see Definition 2.8 below for the precise formulation).

#### Theorem 1.2

Let $p\in [1,\infty )$. Assume that (A) or (B) holds and that $(\rho _{t_k})_{k\in \mathbb {N}}$ has maximal rank. If $(u_k)_{k\in \mathbb {N}}\subset L^p(\mathbb {R}^N)$ is such that $u_k\rightarrow u$ in $L^p(\mathbb {R}^N)$ as $k\rightarrow \infty$ for some $u\in L^p(\mathbb {R}^N)$ and $\displaystyle \liminf _{k\rightarrow \infty } {\mathscr {F}}_{t_k,p}(u_k)<\infty$, then $u\in {\mathcal {S}^{p}}(\mathbb {R}^N)$.

When the conclusion of Theorem 1.2 holds—that is, when the finiteness of the $\liminf$ of the functionals along an $L^p$ convergent sequence implies that the limit function belongs to some subspace ${\mathcal {X}}^p(\mathbb {R}^N)$ of $L^p(\mathbb {R}^N)$ (e.g., ${\mathcal {X}}^p(\mathbb {R}^N)=\mathcal {S}^{p}(\mathbb {R}^N)$)—we say that the functionals $({\mathscr {F}}_{t,p})_{t\in I}$ are *coercive* on ${\mathcal {X}}^p(\mathbb {R}^N)$ (see Definition 2.5 below for a more precise statement).

Any radially symmetric family $(\rho _t)_{t\in I}$ has maximal rank, but non-radially symmetric families with maximal rank are known (examples can be found in [61]). We do not know if the maximal rank condition is also necessary for the conclusion of Theorem 1.2 to hold.

To prove Theorems 1.1 and 1.2, we mix the approaches of [34, 51, 61] in a new fashion.

As in the proof of [34, Th. 1.2], the sufficiency part consists in showing that (1.4) yields the pointwise and $\Gamma$-convergence of $({\mathscr {F}}_{t,p})_{t\in I}$ as $t\rightarrow 0^+$ (up to subsequences) to

1.5 $$\begin{aligned} {\mathscr {G}}^{\mu ,\nu }_p(u) = \int _{{\mathbb {S}}^{N-1}} \Vert \sigma \cdot Du\Vert _{L^p}^p\,\textrm{d}\mu (\sigma ) + \int _{\mathbb {R}^N\setminus \left\{ 0\right\} } \frac{\Vert u(\cdot +z)-u\Vert _{L^p}^p}{|z|^p}\,\textrm{d}\nu (z)\quad \quad \end{aligned}$$

for all $u\in {\mathcal {S}^{p}}(\mathbb {R}^N)$, where $\mu \in {\mathscr {M}}^+({\mathbb {S}}^{N-1})$ and $\nu \in {\mathscr {M}}^+(\mathbb {R}^N)$ are two non-negative finite Radon measures depending on $(\rho _t)_{t\in I}$ only (see Theorem 3.1 for the precise statement). Loosely speaking, the measure $\mu$ is given by (up to subsequences)

1.6 $$\begin{aligned} \mu (E) = \lim _{\delta \rightarrow 0^+} \lim _{t\rightarrow 0^+} \int _E\left( \int _0^\delta \rho _t(\sigma r)\,r^{N-1}\,\textrm{d}r \right) \,\textrm{d}{\mathscr {H}}^{N-1}(\sigma ) \end{aligned}$$

for every Borel set $E\subset {\mathbb {S}}^{N-1}$, while the measure $\nu$ is (up to subsequences) the $\hbox {weak}^\star$ limit of the family $(\rho _t{\mathscr {L}}^N)_{t\in I}$ as $t\rightarrow 0^+$. Consequently, due to (1.5), in order to achieve (1.2), the measure $\nu$ must be supported on $\left\{ 0\right\}$; that is, $\nu =\alpha \delta _0$ for some $\alpha \ge 0$. Additionally, thanks to (1.6), as in [51, 61] the maximal rank assumption guarantees that the limit *p*-Dirichlet energy (1.3) bounds the ${\mathcal {S}^{p}}(\mathbb {R}^N)$ seminorm, yielding Theorem 1.2. To prove the convergence to ${\mathscr {G}}_p^{\mu ,\nu }$, we revise the line of [34] replacing Fourier transform techniques with some plain arguments invoking basic properties of ${\mathcal {S}^{p}}(\mathbb {R}^N)$ functions.

The proof of the necessary part differs from the one of [34] and combines three ingredients. We first show that the validity of (1.2) for some $\mu \in {\mathscr {M}}^+({\mathbb {S}}^{N-1})$ implies (1.4) by testing (1.2) on suitably chosen compactly supported Lipschitz functions. This, in turn, implies that $({\mathscr {F}}_{t,p})_{t\in I}$ converges to ${\mathscr {G}}_p^{\widetilde{\mu },\nu }$ as $t\rightarrow 0^+$ for some $\widetilde{\mu }\in {\mathscr {M}}^+({\mathbb {S}}^{N-1})$ and $\nu \in {\mathscr {M}}^+(\mathbb {R}^N)$ as above. We hence conclude the proof by showing that, if ${\mathscr {G}}_p^{\mu ,0}(u)={\mathscr {G}}_p^{\widetilde{\mu },\nu }(u)$ for all $u\in {\mathcal {S}^{p}}(\mathbb {R}^N)$, then $\nu =\alpha \delta _0$ for some $\alpha \ge 0$ via a scaling argument.

### Compactness

A further research line concerns equicoercivity properties of the functionals (1.1). As is well-known, for $N\ge 2$, the radial symmetry of the family $(\rho _t)_{t\in I}$ yields equicoercivity of the functionals (1.1) (that is, compactness of their sublevel sets), see [20, Th. 4], [62, Ths. 1.2 and 1.3] and [6, Th. 4.2]. If $N=1$, then additional conditions must be imposed due to counterexamples [20, 62].

To the best of our knowledge, no equicoercivity result is available for non-radially symmetric families. Our second main result, inspired by [15, Th. 3.5], partially fills this gap and yields quite flexible *sufficient* conditions on the possibly non-radially symmetric family $(\rho _t)_{t\in I}$ which ensure the equicoercivity of the functionals in (1.1).

In more precise terms, given $p\in [1,\infty )$, we consider

1.7 $$\begin{aligned} \rho _t(x) = \frac{|x|^p K_t(x)}{\phi _{K,\beta ,p}(t)}, \quad \text {for}\ x\in \mathbb {R}^N\ \text {and}\ t\in I, \end{aligned}$$

where $(K_t)_{t\in \mathbb {N}}$ is given by

$$\begin{aligned} K_t(x) = \beta (t)^N K(\beta (t)x), \quad \text {for a.e.}\ x\in \mathbb {R}^N\ \text {and}\ t\in I \end{aligned}$$

for some non-negative function $K\not \equiv 0$ such that $|\cdot |^p\,K\in L^1_\textrm{loc}(\mathbb {R}^N)$ and a Borel function $\beta :I\rightarrow (0,\infty )$. Moreover, in (1.7), we have set that

$$\begin{aligned} \phi _{K,\beta ,p}(t) =\frac{m_{K,p}(\beta (t))}{\beta (t)^p} \quad \text {for all}\ t\in I, \end{aligned}$$

where $m_{K,p}:[0,\infty )\rightarrow [0,\infty )$ is defined as

$$\begin{aligned} m_{K,p}(R)=\int _{B_R}|x|^p\,K(x)\,\textrm{d}x \quad \text {for all}\ R>0. \end{aligned}$$

We refer to Sect. 4.1 for a more detailed description of the family in (1.7).

With the above notation in force, our result can be stated as follows (see Definition 4.3 for the notion of *local precompactness*):

#### Theorem 1.3

With the above notation in force, assume that

$$\begin{aligned} |\cdot |^p\,K\in L^1(\mathbb {R}^N) \quad \text {and} \quad \lim _{t\rightarrow 0^+}\beta (t)=\infty . \end{aligned}$$

If $(t_k)_{k\in \mathbb {N}}\subset I$ is infinitesimal and $(u_k)_{k\in \mathbb {N}}\subset L^p(\mathbb {R}^N)$ is such that

$$\begin{aligned} \sup _{k\in \mathbb {N}} \big ( \Vert u_k\Vert _{L^p} + {\mathscr {F}}_{t_k,p}(u_k) \big )<\infty , \end{aligned}$$

then $(u_k)_{k\in \mathbb {N}}$ is locally precompact in $L^p(\mathbb {R}^N)$ and any of its $L^p_\textrm{loc}(\mathbb {R}^N)$ limits is in ${\mathcal {S}^{p}}(\mathbb {R}^N)$.

The proof of Theorem 1.3 generalizes the strategy in [15] to any $p\in [1,\infty )$. The core idea is to show that, for each $u\in L^p(\mathbb {R}^N)$ and $t>0$, there exists $v_t\in {\mathcal {S}^{p}}(\mathbb {R}^N)$ such that

$$\begin{aligned} \Vert v_t-u\Vert _{L^p} \le C_{K,p} \, {\mathscr {F}}^K_{t,p}(u) \, \beta (t)^{-p} \quad \text {and} \quad \Vert \nabla v_t\Vert _{L^p} \le C_{K,p} \,{\mathscr {F}}^K_{t,p}(u), \end{aligned}$$

where $C_{K,p}>0$ depends on *K* and *p* only (see Proposition 4.4 for the precise statement).

### Non-local Limit Energies

The convergence of $({\mathscr {F}}_{t,p})_{t \in I}$ to the functional ${\mathscr {G}}_p^{\mu ,\nu }$ in (1.5) as $t \rightarrow 0^+$ can be seen as a non-local-to-non-local convergence result. Naturally, one may ask whether the stability of the non-local nature of the functionals also occurs for functions with lower regularity. A similar behavior was, in fact, observed in [2, Th. 1.1(iii)] for characteristic functions of bounded sets with finite (local or non-local) perimeter.

Our next main result aims to provide a deeper understanding of this non-local stability, thereby generalizing [2]. Here and below, given $p\in [1,\infty )$ and a measurable function $\kappa :\mathbb {R}^N\rightarrow [0,\infty ]$, we consider the non-local Sobolev space

$$\begin{aligned} W^{\kappa ,p}(\mathbb {R}^N) = \left\{ u\in L^p(\mathbb {R}^N): [u]_{W^{\kappa ,p}}<\infty \right\} , \end{aligned}$$

where the non-local seminorm is defined by letting

$$\begin{aligned} {[}u]_{W^{\kappa ,p}} = \left( \int _{\mathbb {R}^N}\int _{\mathbb {R}^N}|u(x)-u(y)|^p\,\kappa (x-y)\,\textrm{d}x \,\textrm{d}y\right) ^{1/p}. \end{aligned}$$

We refer, e.g., to [17, 38] for more details on the space $W^{\kappa ,p}(\mathbb {R}^N)$. Here, we just observe that the *fractional Sobolev–Slobodeckij space* $W^{s,p}(\mathbb {R}^N)$, with $s\in (0,1)$ and $p\in [1,\infty )$, corresponds to the choice $\kappa (z)=|z|^{-N-sp}$ for all $z\in \mathbb {R}^N\setminus \left\{ 0\right\}$.

#### Theorem 1.4

Let $p\in [1,\infty )$ and $(\rho _t)_{t\in I}\subset L^1_\textrm{loc}(\mathbb {R}^N)$. Assume that there exist $C>0$ and a measurable function $\kappa :\mathbb {R}^N\rightarrow [0,\infty ]$ such that

1.8 $$\begin{aligned} \frac{\rho _t(z)}{|z|^p} \le C\kappa (z) \quad \text {for all}\ t\in I\ \text {and a.e.}\ z\in \mathbb {R}^N \end{aligned}$$

and

1.9 $$\begin{aligned} \lim _{t\rightarrow 0^+}\frac{\rho _t(z)}{|z|^p}=\kappa (z) \quad \text {for a.e.}\ z\in \mathbb {R}^N. \end{aligned}$$

Then, the limit

$$\begin{aligned} \lim _{t\rightarrow 0^+} {\mathscr {F}}_{t,p}(u) = [u]_{W^{\kappa ,p}}^p, \quad \text {for}\ u\in W^{\kappa ,p}(\mathbb {R}^N), \end{aligned}$$

holds in the pointwise sense and in the $\Gamma$-sense with respect to the $L^p$ topology, and the functionals $({\mathscr {F}}_{t,p})_{t\in I}$ are coercive on $W^{\kappa ,p}(\mathbb {R}^N)$.

As a natural analogue of Theorem 1.3 in this setting, we now complement Theorem 1.4 with the following compactness result:

#### Theorem 1.5

Let $p\in [1,\infty )$ and $(\rho _t)_{t\in I}\subset L^1_\textrm{loc}(\mathbb {R}^N)$. Assume that, for every $\varepsilon >0$, there exists $\delta \in (0,1]$ such that

1.10 $$\begin{aligned} \frac{\rho _t(z)}{|z|^p} \ge \frac{1}{\varepsilon \delta ^N} \quad \text {for a.e.}\ z\in B_\delta \ \text {and every}\ t\in (0,\delta ). \end{aligned}$$

If $(t_k)_{k\in \mathbb {N}}\subset I$ is infinitesimal and $(u_k)_{k\in \mathbb {N}}\subset L^p(\mathbb {R}^N)$ is such that

$$\begin{aligned} \sup _{k\in \mathbb {N}} \big ( \Vert u_k\Vert _{L^p} + {\mathscr {F}}_{t_k,p}(u_k) \big )<\infty , \end{aligned}$$

then $(u_k)_{k\in \mathbb {N}}$ is locally precompact in $L^p(\mathbb {R}^N)$ and any of its $L^p_\textrm{loc}(\mathbb {R}^N)$ limits is in $W^{\kappa ,p}(\mathbb {R}^N)$, where $\kappa :\mathbb {R}^N\rightarrow [0,\infty ]$ is given by

$$\begin{aligned} \kappa (z)=\liminf _{t\rightarrow 0^+}\frac{\rho _t(z)}{|z|^p} \quad \text {for a.e. }\ z\in \mathbb {R}^N. \end{aligned}$$

Theorem 1.4 relies on an application of the Dominated Convergence Theorem and Fatou’s Lemma, exploiting the limits (1.8) and (1.9). Theorem 1.5, instead, is a consequence of the Fréchet–Kolmogorov Compactness Theorem, since the bound (1.10) allows to quantitatively control the $L^p$ distance between functions and their smoothed versions.

The assumptions of Theorems 1.4 and 1.5 are naturally motivated by the application of these results to families induced by *fractional* and *nonlocal heat kernels*; see Theorem 1.7 (in the case $2s < p$) and Theorem 6.2 (in the case $\alpha \in {\mathscr {C}}_p$) below. Although these assumptions are broad enough to cover all the applications considered in this paper, we do not claim that they are sharp, and we leave the analysis of sharp conditions for future work. Nevertheless, we refer to [66] for generalizations of these results.

### Asymptotics of Heat-Type Energies

We apply our results to study the asymptotic behavior of energies induced by heat-type kernels.

Our first main result in this direction concerns the classical *heat semigroup* $(\textsf{H}_t)_{t>0}$, see Sect. 6.1 for the precise definition.

#### Theorem 1.6

If $p\in [1,\infty )$, then the limit

1.11 $$\begin{aligned} \begin{aligned} \lim _{t\rightarrow 0^+} {t^{-\frac{p}{2}}} \int _{\mathbb {R}^N}\textsf{H}_t(|u-u(x)|^p)(x)\,\text {d}x = \frac{2\Gamma (p)}{\Gamma (p/2)} \, \Vert Du\Vert _{L^p} ^p, \quad \text{ for }\ u\in {\mathcal {S}^{p}}(\mathbb {R}^N)\quad \quad \end{aligned}\nonumber \\ \end{aligned}$$

holds in the pointwise and $\Gamma$-sense with respect to the $L^p$ topology, and the functionals on the left-hand side are coercive on ${\mathcal {S}^{p}}(\mathbb {R}^N)$. Moreover, if $(t_k)_{k\in \mathbb {N}}\subset I$ is infinitesimal and $(u_k)_{k\in \mathbb {N}}\subset L^p(\mathbb {R}^N)$ is such that

$$\begin{aligned} \liminf _{k\rightarrow \infty } {t^{-\frac{p}{2}}_k} \int _{\mathbb {R}^N}\textsf{H}_{t_k}(|u_k-u_k(x)|^p)(x)\,\textrm{d}x<\infty , \end{aligned}$$

then $(u_k)_{k\in \mathbb {N}}$ is locally precompact in $L^p(\mathbb {R}^N)$ and any of its $L^p_\textrm{loc}(\mathbb {R}^N)$ limits is in ${\mathcal {S}^{p}}(\mathbb {R}^N)$.

The pointwise limit in Theorem 1.6 and its link with the BBM formula are already known, see [42, Th. B] and the related discussion for example. However, we were not able to trace the $\Gamma$-convergence and compactness parts of Theorem 1.6 in the literature.

It is worth mentioning that formula (1.11) plays a central role in the study of small time asymptotics for the heat semigroup. For $p=1$ and $u=\chi _E\in BV(\mathbb {R}^N)$, the limit in (1.11) can be rewritten as

1.12 $$\begin{aligned} Q_E(t){:}{=}\int _E\textsf{H}_t\chi _E\,\textrm{d}x = |E| - \frac{1}{\sqrt{\pi }}\,P(E)\cdot \sqrt{t} + o(\sqrt{t}) \quad \text {as}\ t\rightarrow 0^+,\qquad \end{aligned}$$

which is the so-called (*relative*) *heat content* of the set *E*. From a physical perspective, if *E* is a container that is perfectly insulated at time $t=0$, then $Q_E(t)$ measures the amount of heat that remains inside *E* at time $t>0$.

The study of the short-time behavior of heat-semigroup energies originated from De Giorgi’s seminal work [35], and later expanded across various settings, including not only Euclidean spaces [11, 50, 58, 63, 67], but also Riemannian manifolds [8], Carnot groups [42], sub-Riemannian manifolds [3, 28], and $\textsf{RCD}$ metric-measure spaces [22]. For smooth sets *E*, the asymptotic expansion in (1.12) continues in powers of $\sqrt{t}$, with coefficients encoding fundamental geometric features of *E*, such as mean curvature.

Our second main result is the fractional counterpart of Theorem 1.6, dealing with the *fractional heat semigroup* $(\textsf{H}_t^s)_{t>0}$, with $s\in (0,1)$; see Sect. 6.2 for the precise definition.

#### Theorem 1.7

Given $p\in [1,\infty )$ and $s\in (0,1)$, let $\psi _{s,p}:I\rightarrow [0,\infty )$ be defined as

$$\begin{aligned} \psi _{s,p}(t) = {\left\{ \begin{array}{ll} t^{\frac{p}{2s}} &  \text {if}\ 2s>p, \\ t|\log t| &  \text {if}\ 2s=p, \\ t &  \text {if}\ 2s<p \end{array}\right. } \end{aligned}$$

for all $t\in I$. The limits

$$\begin{aligned} \lim _{t\rightarrow 0^+} \int _{\mathbb {R}^N}\frac{\textsf{H}_t^s(|u-u(x)|^p)(x)}{\psi _{s,p}(t)}\,\textrm{d}x = {\left\{ \begin{array}{ll} \displaystyle \frac{\Gamma \left( 1-\frac{p}{2s}\right) }{\Gamma \left( 1-\frac{p}{2}\right) } \,\frac{2\Gamma (p)}{\Gamma (p/2)} \, \Vert Du\Vert _{L^p}^p &  \text {in}\ {\mathcal {S}^{p}}(\mathbb {R}^N)\ \text {if}\ 2s\ge p, \\[5ex] \displaystyle \frac{s\,4^s}{\pi ^{\frac{N}{2}}} \, \frac{\Gamma \left( \frac{N}{2}+s\right) }{\Gamma (1-s)} \, [u]_{W^{2s,\frac{p}{2s}}}^p &  \text {in}\ W^{2s,\frac{p}{2s}}(\mathbb {R}^N)\ \text {if}\ 2s<p, \end{array}\right. } \end{aligned}$$

hold in the pointwise and $\Gamma$-sense with respect to the $L^p$ topology, and all the functionals on the left-hand sides are coercive on the respective spaces. Moreover, if $(t_k)_{k\in \mathbb {N}}\subset I$ is infinitesimal and $(u_k)_{k\in \mathbb {N}}\subset L^p(\mathbb {R}^N)$ is such that

$$\begin{aligned} \liminf _{k\rightarrow \infty } \int _{\mathbb {R}^N}\frac{\textsf{H}_{t_k}^s(|u_k-u_k(x)|^p)(x)}{\psi _{s,p}(t_k)}\,\textrm{d}x<\infty , \end{aligned}$$

then $(u_k)_{k\in \mathbb {N}}$ is locally precompact in $L^p(\mathbb {R}^N)$ and any of its $L^p_\textrm{loc}(\mathbb {R}^N)$ limits is in ${\mathcal {S}^{p}}(\mathbb {R}^N)$ if $2s\ge p$ and in $W^{2s,\frac{p}{2s}}(\mathbb {R}^N)$ if $2s<p$.

For $p=1$ and for characteristic functions of sets only, the pointwise limits in Theorem 1.7 were obtained in [2, Th. 4.1] under the additional assumption that the sets under consideration are bounded, while the $\Gamma$-limits were established in the recent work [47]. The strategies of proof in [2, 47] are both different from our approach. We refer to Remarks 6.1 and 6.3 below for further comments on the relation between our work and [2, 47].

In fact, we can prove a more general version of Theorem 1.7, generalizing [2, Th. 1.1]. For the precise statement, which is more involved, we refer to Theorem 6.2 below.

### Other Approaches in Hilbert Spaces

In the Hilbertian case $p=2$, Theorems 1.6 and 1.7 can be achieved in alternative and more general ways.

Let ${\mathcal {H}}$ be a Hilbert space, $(\textsf{H}_t)_{t\ge 0}$ be a *strongly continuous semigroup of symmetric operators* on ${\mathcal {H}}$, and $L$ be the (*infinitesimal*) *generator* of $(\textsf{H}_t)_{t\ge 0}$ with domain ${\mathcal {D}}(L)\subset {\mathcal {H}}$. In this setting, the *semigroup content* of $u\in {\mathcal {H}}$ is defined as the map

$$\begin{aligned} {}[0,\infty )\ni t\mapsto {{\mathbb {H}}}_t(u){:}{=}(\textsf{H}_tu,u)_{{\mathcal {H}}}. \end{aligned}$$

With this notation in force, we can state our last main result.

#### Theorem 1.8

Let ${\mathcal {H}}$, $(\textsf{H}_t)_{t\ge 0}$ and $L$ be as above. The following hold:

i if $$u\in {\mathcal {D}}(L)$$, then $$\displaystyle \limsup _{t\rightarrow 0^+} \frac{{{\mathbb {H}}}_0(u)-{{\mathbb {H}}}_t(u)}{t} \le (-Lu,u)_{{\mathcal {H}}}$$;
ii if $(t_k)_{k\in \mathbb {N}}\subset (0,\infty )$ is infinitesimal and $(u_k)_{k\in \mathbb {N}}\subset {\mathcal {H}}$ is such that $u_k\rightarrow u$ in ${\mathcal {H}}$ as $k\rightarrow \infty$ for some $u\in {\mathcal {D}}(L)$, then
  $$\begin{aligned} \liminf _{k\rightarrow \infty } \frac{{{\mathbb {H}}}_0(u_k)-{{\mathbb {H}}}_{t_k}(u_k)}{t_k} \ge (-Lu,u)_{{\mathcal {H}}}. \end{aligned}$$

As a consequence, the functionals $u\mapsto \frac{{{\mathbb {H}}}_0(u)-{{\mathbb {H}}}_{t}(u)}{t}$ converge to $u\mapsto (-Lu,u)_{{\mathcal {H}}}$ on ${\mathcal {H}}$ as $t\rightarrow 0^+$ pointwise and in the $\Gamma$-sense with respect to the strong topology in ${\mathcal {H}}$.

The proof of Theorem 1.6 exploits some elementary arguments involving the spectral representation of the non-negative operator $-L$. We observe that, in the case ${\mathcal {H}}=L^2(\mathbb {R}^N)$ and $L=-(-\Delta )^s$ with $s\in (0,1]$, Theorem 1.8 covers the case $p=2$ in Theorems 1.6 and 1.7. Actually, if ${\mathcal {H}}=L^2(\mathbb {R}^N)$ and the semigroup of operators $(\textsf{H}_t)_{t\ge 0}$ is given by

$$\begin{aligned} (\textsf{H}_t u,v)_{L^2} = \int _{\mathbb {R}^N}e^{-\lambda (\xi )t}\,{\hat{u}}(\xi )\cdot \overline{{\hat{v}}(\xi )}\,\textrm{d}\xi \end{aligned}$$

for all $t\ge 0$ and $u,v\in L^2(\mathbb {R}^N)$, where $\lambda :\mathbb {R}^N\rightarrow [0,\infty ]$ is a measurable function (in the aforementioned cases, $\lambda (\xi )=(2\pi |\xi |)^{2s}$ for $\xi \in \mathbb {R}^N$ and $s\in (0,1]$), then a different and simpler approach via Fourier transform is also possible, see Sect. 7.2 for more details.

### Organization of the Paper

The rest of the paper is organized as follows: in Sect. 2, we provide the main notation and the basic results used throughout the paper. In Sect. 3, we deal with the sharp conditions for the BBM formula in Theorem 1.1. In Sect. 4, we specialize the BBM formula to the family of kernels (1.7) and we prove the compactness criterion stated in Theorem 1.3. In Sect. 5, we treat non-local-to-non-local results, proving Theorems 1.4 and 1.5. In Sect. 6, we apply our theorems to the study of energies induced by heat-type kernels, both in the local and in the non-local setting, proving Theorems 1.6 and 1.7. Finally, in Sect. 7, we detail the proof of Theorem 1.8 and present an alternative proof of heat content asymptotics in $L^2(\mathbb {R}^N)$ via Fourier transform.

## Preliminaries

### General Notation

We let $N\in \mathbb {N}$ and ${\mathbb {S}}^{N-1}=\left\{ x\in \mathbb {R}^N:|x|=1\right\}$ be the $(N-1)$-dimensional unit sphere in $\mathbb {R}^N$.

We let ${\mathscr {L}}^N$ be the *N*-dimensional Lebesgue measure in $\mathbb {R}^N$ and we let ${\mathscr {H}}^s$ be the *s*-dimensional Hausdorff measure in $\mathbb {R}^N$, with $s\in [0,N]$. All sets and functions are assumed to be Lebesgue measurable. We use the shorthand $|E|={\mathscr {L}}^N(E)$ for $E\subset \mathbb {R}^N$.

Given a non-empty set $X\subset \mathbb {R}^N$, we let *C*(*X*) and $\operatorname {Lip}(X)$ be the spaces of continuous and Lipschitz continuous functions on *X*, respectively. As customary, we let $C_c(X)$ and $\operatorname {Lip}_c(X)$ be their subsets of compactly supported functions, respectively. If *X* is open, then we also let $C^\infty (X)$ and $C^\infty _c(X)$ be the spaces of smooth functions and of smooth functions with compact support on *X*, respectively.

### Radon Measures

Let $X\subset \mathbb {R}^N$ be a non-empty set. We let ${\mathscr {M}}(X)$ and ${\mathscr {M}}_\textrm{loc}(X)$ be the spaces of finite and locally finite signed Radon measures on *X*, respectively. We also let ${\mathscr {M}}^+(X)$ and ${\mathscr {M}}_\textrm{loc}^+(X)$ be their subsets of non-negative measures, respectively.

By the Riesz Representation Theorem, ${\mathscr {M}}_\textrm{loc}(X)$ can be identified as the dual of $C_c(X)$, endowed with local uniform convergence. Thus, we say that $(\mu _k)_{k\in \mathbb {N}}\subset {\mathscr {M}}_\textrm{loc}(X)$ converges to $\mu \in {\mathscr {M}}_\textrm{loc}(X)$ in the (*local*) ${ weak}^\star$ *sense*, and we write $\mu _k\overset{\star }{\rightharpoonup }\mu$ in ${\mathscr {M}}_\textrm{loc}(X)$ as $k\rightarrow \infty$, if

2.1 $$\begin{aligned} \lim _{k\rightarrow \infty } \int _Xf\,\textrm{d}\mu _k = \int _Xf\,\textrm{d}\mu \quad \text {for every}\ f\in C_c(X). \end{aligned}$$

We recall that, if $\mu _k\overset{\star }{\rightharpoonup }\mu$ in ${\mathscr {M}}_\textrm{loc}(X)$ as $k\rightarrow \infty$, then (2.1) actually holds for every bounded Borel function $f:X\rightarrow \mathbb {R}$ with compact support such that the set of its discontinuity points is $\mu$-negligible. Consequently, if *X* is compact and $\mu _k\overset{\star }{\rightharpoonup }\mu$ in ${\mathscr {M}}_\textrm{loc}(X)$ as $k\rightarrow \infty$, then (2.1) holds for every $f\in C(X)$. Therefore, in this case, we simply write $\mu _k\overset{\star }{\rightharpoonup }\mu$ in ${\mathscr {M}}(X)$ as $k\rightarrow \infty$. See [9] for a more detailed discussion.

For future convenience, we recall the following result, which corresponds to [61, Lem. 6]:

#### Lemma 2.1

Let $\mu \in {\mathscr {M}}^+({\mathbb {S}}^{N-1})$ and let $\Theta _\mu \in C(\mathbb {R}^N)$ be defined as

2.2 $$\begin{aligned} \Theta _\mu (v) = \int _{{\mathbb {S}}^{N-1}}|v\cdot \sigma |\,\textrm{d}\mu (\sigma ), \quad \text {for every}\ v\in \mathbb {R}^N. \end{aligned}$$

Then, $\min _{{\mathbb {S}}^{N-1}}\Theta _\mu >0$ if and only if $\operatorname {span}(\operatorname {supp}\mu )=\mathbb {R}^N$.

### Sobolev and *BV* Spaces

For $p\in [1,\infty )$, we let

$$\begin{aligned} {\mathcal {S}^{p}}(\mathbb {R}^N) = {\left\{ \begin{array}{ll} W^{1,p}(\mathbb {R}^N) &  \text {for}\ p>1 \\ BV(\mathbb {R}^N) &  \text {for}\ p=1. \end{array}\right. } \end{aligned}$$

As customary, *Du* denotes the distributional gradient of $u\in {\mathcal {S}^{p}}(\mathbb {R}^N)$. In particular, if $p=1$, then *Du* may be a finite Radon measure on $\mathbb {R}^N$. We endow ${\mathcal {S}^{p}}(\mathbb {R}^N)$ with the norm

$$\begin{aligned} \Vert u\Vert _{{\mathcal {S}^{p}}(\mathbb {R}^N)} = \left( \Vert u\Vert _{L^p}^p+\Vert Du\Vert _{L^p}^p\right) ^{1/p}, \quad \text {for}\ u\in {\mathcal {S}^{p}}(\mathbb {R}^N), \end{aligned}$$

where, as customary, we have set that

2.3 $$\begin{aligned} \Vert Du\Vert _{L^p}^p = {\left\{ \begin{array}{ll} \displaystyle \int _{\mathbb {R}^N}|Du(x)|^p\,\textrm{d}x &  \text {for}\ p>1, \\ |Du|(\mathbb {R}^N) &  \text {for}\ p=1. \end{array}\right. } \end{aligned}$$

We recall the following simple result, whose proof is omitted:

#### Lemma 2.2

Let $p\in [1,\infty )$ and $z\in \mathbb {R}^N$. The following hold:

i if $$u\in \mathcal {S}^{p}(\mathbb {R}^N)$$, then $$\Vert u(\,\cdot +z)-u\Vert _{L^p}\le \Vert z\cdot Du\Vert _{L^p}$$;
ii if $u\in W^{2,p}(\mathbb {R}^N)$, then $\left| \Vert u(\,\cdot +z)-u\Vert _{L^p} - \Vert z\cdot Du\Vert _{L^p} \right| \le \frac{|z|^2}{2}\,\Vert D^2u\Vert _{L^p}$.

### Non-local Sobolev Spaces

Given $p\in [1,\infty )$ and a non-negative measurable function $\kappa :\mathbb {R}^N\rightarrow [0,\infty ]$, we let

$$\begin{aligned} W^{\kappa ,p}(\mathbb {R}^N) = \left\{ u\in L^p(\mathbb {R}^N): [u]_{W^{\kappa ,p}}<\infty \right\} , \end{aligned}$$

where

$$\begin{aligned} {[}u]_{W^{\kappa ,p}} = \left( \int _{\mathbb {R}^N}\Vert u(\cdot +z)-u\Vert _{L^p}^p\,\kappa (z)\,\textrm{d}z\right) ^{1/p} \end{aligned}$$

for $u\in L^p(\mathbb {R}^N)$. We note that $W^{\kappa ,p}(\mathbb {R}^N)$, endowed with the norm

$$\begin{aligned} \Vert u\Vert _{W^{\kappa ,p}} = \left( \Vert u\Vert _{L^p}^p+[u]_{W^{\kappa ,p}}^p\right) ^{1/p}, \quad \text {for}\ u\in W^{\kappa ,p}(\mathbb {R}^N), \end{aligned}$$

is a Banach space. We refer to [17, 38] for a more detailed presentation. Here we only mention that the *fractional Sobolev–Slobodeckij space* $W^{s,p}(\mathbb {R}^N)$, with $s\in (0,1)$ and $p\in [1,\infty )$, corresponds to the choice $\kappa (z)=|z|^{-N-sp}$ for all $z\in \mathbb {R}^N\setminus \left\{ 0\right\}$.

### Convergence of Functionals on $L^p(\mathbb {R}^N)$

Let $p\in [1,\infty )$ and let ${\mathcal {X}}^p(\mathbb {R}^N)\subset L^p(\mathbb {R}^N)$. Given $F_k,G:L^p(\mathbb {R}^N)\rightarrow [0,\infty ]$, $k\in \mathbb {N}$, we adopt the following terminology. For a complete description of $\Gamma$*-convergence*, we refer to the monographs [21, 31].

#### Definition 2.3

(*Pointwise convergence*) We say that $(F_k)_{k\in \mathbb {N}}$ converges to *G* on ${\mathcal {X}}^p(\mathbb {R}^N)$ as $k\rightarrow \infty$ in the *pointwise sense* if $\displaystyle \lim _{k\rightarrow \infty }F_k(u)=G(u)$ for every $u\in {\mathcal {X}}^p(\mathbb {R}^N)$.

#### Definition 2.4

($\Gamma$-*convergence*) We say that $(F_k)_{k\in \mathbb {N}}$ converges to *G* on ${\mathcal {X}}^p(\mathbb {R}^N)$ as $k\rightarrow \infty$ in the $\Gamma$*-sense with respect to the* $L^p$ *topology* if the following two properties hold:

- ($\Gamma$-$\liminf$) if $(u_k)_{k\in \mathbb {N}}\subset L^p(\mathbb {R}^N)$ is such that $u_k\rightarrow u$ in $L^p(\mathbb {R}^N)$ as $k\rightarrow \infty$ for some $u\in {\mathcal {X}}^p(\mathbb {R}^N)$, then $\displaystyle \liminf _{k\rightarrow \infty } F_k(u_k) \ge G(u)$;
- ($\Gamma$-$\limsup$) if $u\in {\mathcal {X}}^p(\mathbb {R}^N)$, then there exists $(u_k)_{k\in \mathbb {N}}\subset {\mathcal {X}}^p(\mathbb {R}^N)$ such that $u_k\rightarrow u$ in $L^p(\mathbb {R}^N)$ as $k\rightarrow \infty$ and $\displaystyle \limsup _{k\rightarrow \infty }F_k(u_k)\le G(u)$.

#### Definition 2.5

(*Coerciveness*) We say that $(F_k)_{k\in \mathbb {N}}$ is *coercive* on ${\mathcal {X}}^p(\mathbb {R}^N)$ if, whenever $(u_k)_{k\in \mathbb {N}}\subset L^p(\mathbb {R}^N)$ is such that $u_k\rightarrow u$ for some $u\in L^p(\mathbb {R}^N)$ as $k\rightarrow \infty$ and

$$\begin{aligned} \liminf _{k\rightarrow \infty } F_k(u_k)<\infty , \end{aligned}$$

then $$u\in {\mathcal {X}}^p(\mathbb {R}^N)$$.

### Family of Kernels

Throughout the paper, we let $I=(0,1)$. We let $(\rho _t)_{t\in I}\subset L^1_\textrm{loc}(\mathbb {R}^N)$ be a family of non-negative kernels, $\rho _t\ge 0$ for every $t\in I$. The next result generalizes [34, Lem. 5.2]. We briefly detail its proof for the convenience of the reader.

#### Lemma 2.6

Let $p\in [1,\infty )$ and $J\subset I$ be such that $0\in {\overline{J}}$. The following are equivalent:

i $$\displaystyle \sup _{R>0} \limsup _{t\in J,\,t\rightarrow 0^+} R^p\int _{\mathbb {R}^N}\frac{\rho _t(z)}{R^p+|z|^p}\,\textrm{d}z<\infty $$;
ii $$\displaystyle \sup _{R>0} \left[ \limsup _{t\in J,\,t\rightarrow 0^+} \int _{B_R}\rho _t(z)\,\textrm{d}z + \limsup _{t\in J,\,t\rightarrow 0^+} R^p\int _{B_R^c}\frac{\rho _t(z)}{|z|^p}\,\textrm{d}z \right] <\infty $$;
iii there exists $R_0>0$ such that
  $$\begin{aligned} \limsup _{t\in J,\,t\rightarrow 0^+}\int _{B_{R_0}}\rho _t(z)\,\textrm{d}z + \sup _{R>R_0} \limsup _{t\in J,\,t\rightarrow 0^+} R^p\int _{B_R^c}\frac{\rho _t(z)}{|z|^p}\,\textrm{d}z<\infty ; \end{aligned}$$
iv $$\displaystyle \sup _{R>0}\limsup _{t\in J,\,t\rightarrow 0^+}\int _{B_R}\rho _t(z)\,\textrm{d}z<\infty $$ and
  $$\begin{aligned} \limsup _{t\in J,\,t\rightarrow 0^+}\int _{B_R^c}\frac{\rho _t(z)}{|z|^p}\,\textrm{d}z=0 \quad \text {for all}\ R>0; \end{aligned}$$
v $$\displaystyle \limsup _{t\in J,\,t\rightarrow 0^+}\int _{\mathbb {R}^N}(1\wedge |z|^{-p})\,\rho _t(z)\,\textrm{d}z<\infty $$ and
  $$\begin{aligned} \limsup _{t\in J,\,t\rightarrow 0^+}\int _{B_R^c}(1\wedge |z|^{-p})\,\rho _t(z)\,\textrm{d}z=0 \quad \text {for all}\ R>0. \end{aligned}$$

#### Proof

The equivalence (i)$\iff$(ii) can be proved *verbatim* as in [34, Lem. 5.2]. It is clear that (ii)$\implies$(iv), while the fact that

$$\begin{aligned} \int _{B_R}\rho _t(z)\,\textrm{d}z = \left( \int _{B_{R_0}}+ \int _{B_R\setminus B_{R_0}}\right) \rho _t(z)\,\textrm{d}z \le \int _{B_{R_0}}\rho _t(z)\,\textrm{d}z + R^p \int _{B_R} \frac{\rho _t(z)}{|z|^p}\,\textrm{d}z \end{aligned}$$

for every $R>R_0$ yields that (iv)$\implies$(ii). The implication (ii)$\implies$(iv) is obvious. Conversely, given $R>0$, by the first part of (iv) we can find $M>R$ such that

$$\begin{aligned} \limsup _{t\in J,\,t\rightarrow 0^+}\int _{B_M^c}\frac{\rho _t(z)}{|z|^p}\,\textrm{d}z \le \frac{1}{R^p}, \end{aligned}$$

so that

$$\begin{aligned} \begin{aligned} \limsup _{t\in J,\,t\rightarrow 0^+}\int _{B_R^c}\frac{\rho _t(z)}{|z|^p}\,\textrm{d}z&\le \limsup _{t\in J,\,t\rightarrow 0^+}\int _{B_M^c}\frac{\rho _t(z)}{|z|^p}\,\textrm{d}z + \limsup _{t\in J,\,t\rightarrow 0^+}\int _{B_M\setminus B_R}\frac{\rho _t(z)}{|z|^p}\,\textrm{d}z \\&\le \frac{1}{R^p} + \frac{1}{R^p} \limsup _{t\in J,\,t\rightarrow 0^+}\int _{B_M\setminus B_R}\rho _t(z)\,\textrm{d}z \le \frac{C+1}{R^p}, \end{aligned} \end{aligned}$$

where

$$\begin{aligned} C{:}{=}\sup _{r>0}\limsup _{t\in J,\,t\rightarrow 0^+}\int _{B_r}\rho _t(z)\,\textrm{d}z<\infty , \end{aligned}$$

proving that (iv)$\implies$(ii). The equivalence (v)$\iff$(iv) follows via elementary arguments, so we omit its proof. $\square$

#### Remark 2.7

The equivalence (i)$\iff$(ii) in Lemma 2.6 is proved in [34, Lem. 5.2] for $p=2$. Property (v) in Lemma 2.6 is exploited in [38] for radially symmetric families $(\rho _t)_{t\in I}$ and $p>1$, see [38, Sect. 9.2] for more details. A family complying with any of the conditions in Lemma 2.6 is given by the standard fractional kernels [20, 61], defined as

$$\begin{aligned} \rho _t(z)=\frac{1-t}{|z|^{N-(1-t)p}} \end{aligned}$$

for $$z\in \mathbb {R}^N\setminus \left\{ 0\right\} $$, $$t\in I$$, and $$p\in [1,\infty )$$.

Following [61, Sect. 1.3] and [51, Sect. 1], we introduce the following terminology: from now on, given $\tau \in I$ and $v\in {\mathbb {S}}^{N-1}$, we set

$$\begin{aligned} {\mathcal {C}}_\tau (v) = \left\{ x\in \mathbb {R}^N: x\cdot v\ge (1-\tau )\,|x|\right\} . \end{aligned}$$

#### Definition 2.8

(*Maximal rank*) Let $J\subset I$ be such that $0\in {\overline{J}}$. We say that the family $(\rho _t)_{t\in J}\subset L^1_\textrm{loc}(\mathbb {R}^N)$ has *maximal rank* if there exist $\tau \in I$ and a basis $v_1,\dots , v_N\in {\mathbb {S}}^{N-1}$ of $\mathbb {R}^N$ such that ${\mathcal {C}}_\tau (v_i)\cap {\mathcal {C}}_\tau (v_j)=\left\{ 0\right\}$ for every $i,j\in \left\{ 1,\dots ,N\right\}$ such that $i\ne j$, and

$$\begin{aligned} \inf _{\delta>0} \, \min _{i\in \left\{ 1,\dots ,N\right\} } \, \liminf _{t\in J,\,t\rightarrow 0^+} \int _{B_\delta \,\cap \,{\mathcal {C}}_\tau (v_i)}\rho _t(z)\,\textrm{d}z>0. \end{aligned}$$

This maximal-rank condition is the nonlocal analogue of *uniform ellipticity*, guaranteeing full *N*-dimensional control of all (non-local) partial derivatives: the family of kernels is forced to retain a uniform amount of mass in narrow cones around every basis direction, so it never collapses onto a lower-dimensional subspace.

The (first part of the) next result was implicitly proved across the proof of [34, Prop. 3.2]. For similar results, we refer to [61] and [51, Lem. 2.1].

#### Lemma 2.9

Let $J\subset I$ be such that $0\in {\overline{J}}$. If

$$\begin{aligned} \sup _{R>0}\limsup _{t\in J,\,t\rightarrow 0^+}\int _{B_R}\rho _t(z)\,\textrm{d}z<\infty , \end{aligned}$$

then there exist a countable set $I_0\subset I$, two infinitesimal sequences $(t_k)_{k\in \mathbb {N}}\subset J$ and $(\delta _l)_{l\in \mathbb {N}}\subset I{\setminus } I_0$, and two measures $\mu \in {\mathscr {M}}^+({\mathbb {S}}^{N-1})$ and $\nu \in {\mathscr {M}}^+(\mathbb {R}^N)$ such that

i letting $\nu _k=\rho _{t_k}\,{\mathscr {L}}^N$ for every $k\in \mathbb {N}$, it holds that $\nu _k\overset{\star }{\rightharpoonup }\nu$ in ${\mathscr {M}}_\textrm{loc}(\mathbb {R}^N)$ as $k\rightarrow \infty$;
ii letting $A_l=\left\{ x\in \mathbb {R}^N : \delta _l<|x|<\frac{1}{\delta _l}\right\}$ for every $l\in \mathbb {N}$, it holds that
  $$\begin{aligned} \lim _{k\rightarrow \infty } \int _{A_l} f\,\textrm{d}\nu _k = \int _{A_l}f\,\textrm{d}\nu , \quad \text {for every } l\in \mathbb {N}\text { and } f\in C(\mathbb {R}^N\setminus \left\{ 0\right\} ); \end{aligned}$$
iii letting $\mu _t^\delta \in {\mathscr {M}}^+({\mathbb {S}}^{N-1})$ be given by
  2.4 $$\begin{aligned} \mu _t^\delta (E) = \int _E\left( \int _0^\delta \rho _t(\sigma r)\,r^{N-1}\,\textrm{d}r\right) \,\textrm{d}{\mathscr {H}}^{N-1}(\sigma ) \end{aligned}$$
  for every ${\mathscr {H}}^{N-1}$-measurable set $E\subset {\mathbb {S}}^{N-1}$ and $t,\delta \in I$, it holds that:
  - letting $\mu _k^l=\mu _{t_k}^{\delta _l}$ for every $k,l\in \mathbb {N}$, there exists $(\mu ^l)_{l\in \mathbb {N}}\subset {\mathscr {M}}^+({\mathbb {S}}^{N-1})$ such that $\mu _k^l\overset{\star }{\rightharpoonup }\mu ^l$ in ${\mathscr {M}}({\mathbb {S}}^{N-1})$ as $k\rightarrow \infty$ for every $l\in \mathbb {N}$;
  - the sequence $(\mu ^l)_{l\in \mathbb {N}}\subset {\mathscr {M}}^+({\mathbb {S}}^{N-1})$ is such that $\mu ^l\overset{\star }{\rightharpoonup }\mu$ in ${\mathscr {M}}({\mathbb {S}}^{N-1})$ as $l\rightarrow \infty$.

In addition, if $(\rho _t)_{t\in J}$ has maximal rank, then $\operatorname {span}(\operatorname {supp}\mu )=\mathbb {R}^N$.

In the proof of Theorem 2.9, we will need the following simple result, which can be inferred as in the proof of [51, Lem. 2.1]. (we thus omit its proof).

#### Lemma 2.10

Let $\tau \in I$ and let $v_1,\dots ,v_N\in {\mathbb {S}}^{N-1}$ be a basis of $\mathbb {R}^N$ such that ${\mathcal {C}}_\tau (v_i)\cap {\mathcal {C}}_\tau (v_j)=\left\{ 0\right\}$ for every $i,j\in \left\{ 1,\dots ,N\right\}$ such that $i\ne j$. Then, there exists $c_0>0$ such that, for each $v\in {\mathbb {S}}^{N-1}$, there exists $i_0\in \left\{ 1,\dots ,N\right\}$ such that $|v\cdot \sigma |\ge c_0$ for every $\sigma \in {\mathcal {C}}_\tau (v_{i_0})\cap {\mathbb {S}}^{N-1}$.

#### Proof of Theorem 2.9

Let us set

2.5 $$\begin{aligned} M=\sup _{R>0}\limsup _{t\in J,\,t\rightarrow 0^+}\int _{B_R}\rho _t(z)\,\textrm{d}z<\infty . \end{aligned}$$

We split the proof into three steps.

*Step 1*. Let $\nu _t=\rho _t\,{\mathscr {L}}^N$ for all $t\in J$. Let $(R_k)_{k\in \mathbb {N}}\subset (0,\infty )$ be a strictly increasing and unbounded sequence. Owing to (2.5), we can find a strictly decreasing and infinitesimal sequence $(t_k)_{k\in \mathbb {N}}\subset J$ such that

2.6 $$\begin{aligned} \nu _{t_k}(B_{R_k}) = \int _{B_{R_k}}\rho _{t_k}(z)\,\textrm{d}z \le M+\frac{1}{k} \end{aligned}$$

for each $k\in \mathbb {N}$. By known results (see [9, Th. 1.59] for example), we can find $\nu \in {\mathscr {M}}^+(\mathbb {R}^N)$ such that, up to passing to a non-relabeled subsequence of $(t_k)_{k\in \mathbb {N}}$, $\nu _{t_k}\overset{\star }{\rightharpoonup }\nu$ in ${\mathscr {M}}_\textrm{loc}(\mathbb {R}^N)$ as $k\rightarrow \infty$, and $\nu (\mathbb {R}^N)\le M+1$.

*Step 2*. Let us set $A_\delta =\left\{ x\in \mathbb {R}^N : \delta<|x|<\frac{1}{\delta }\right\}$ for all $\delta \in I$. By definition, the family $(\partial A_\delta )_{\delta \in I}$ consists of pairwise disjoint compact sets in $\mathbb {R}^N$ which are precisely the sets of discontinuity points of the functions $(\chi _{A_\delta })_{\delta \in I}$. We can thus apply [9, Prop. 1.62(b)] (see also the discussion in [9, Ex. 1.63]) and find a countable set $I_0\subset I$ such that $\nu (\partial A_\delta )=0$ for all $\delta \in I\setminus I_0$, where $\nu$ is the measure obtained in Step 1. Since $\nu _{t_k}\overset{\star }{\rightharpoonup }\nu$ in ${\mathscr {M}}_\textrm{loc}(\mathbb {R}^N)$ as $k\rightarrow \infty$ by Step 1 and $\bigcup _{\delta \in I}A_\delta =\mathbb {R}^N{\setminus }\left\{ 0\right\}$ by construction, we hence infer that

$$\begin{aligned} \lim _{k\rightarrow \infty } \int _{A_\delta }f\,\textrm{d}\nu _k = \int _{A_\delta }f\,\textrm{d}\nu \quad \text {for every } \delta \in I\setminus I_0 \text { and } f\in C(\mathbb {R}^N\setminus \left\{ 0\right\} ). \end{aligned}$$

*Step 3*. Now pick any strictly decreasing sequence $(\delta _l)_{l\in \mathbb {N}}\subset I\setminus I_0$ such that $\delta _l\rightarrow 0^+$ as $l\rightarrow \infty$ and consider the measures $(\mu _{t_k}^{\delta _l})_{l,k\in \mathbb {N}}$ as defined in (2.4), where $(t_k)_{k\in \mathbb {N}}$ is as in Step 1. Owing to (2.4) and to (2.6), we get that

$$\begin{aligned} \mu _{t_k}^{\delta _l}({\mathbb {S}}^{N-1}) \le \!\! \int _{{\mathbb {S}}^{N-1}} \left( \int _0^1 \rho _{t_k}(\sigma r)\,r^{N-1}\,\textrm{d}r \right) \,\textrm{d}{\mathscr {H}}^{N-1}(\sigma ) =\!\!\int _{B_1}\rho _{t_k}(z)\,\textrm{d}z \le M+1 \end{aligned}$$

for each $k\in \mathbb {N}$ sufficiently large and each $l\in \mathbb {N}$. Thus, for each $l\in \mathbb {N}$, we can find a subsequence $(t_k^l)_{k\in \mathbb {N}}$ of $(t_k)_{k\in \mathbb {N}}$ and $\mu ^l\in {\mathscr {M}}^+({\mathbb {S}}^{N-1})$ such that $\mu _{t_k^l}^{\delta _l}\overset{\star }{\rightharpoonup }\mu ^l$ in ${\mathscr {M}}({\mathbb {S}}^{N-1})$ as $k\rightarrow \infty$ and $\mu ^l({\mathbb {S}}^{N-1})\le M+1$. By a routine diagonal argument, we can find subsequence $(t_k')_{k\in \mathbb {N}}$ of $(t_k)_{k\in \mathbb {N}}$ such that $\mu _{t_k'}^{\delta _l}\overset{\star }{\rightharpoonup }\mu ^l$ in ${\mathscr {M}}({\mathbb {S}}^{N-1})$ as $k\rightarrow \infty$ for all $l\in \mathbb {N}$. Moreover, we can find a subsequence $(\delta _{l_j})_{j\in \mathbb {N}}$ of $(\delta _l)_{l\in \mathbb {N}}$ and $\mu \in {\mathscr {M}}^+({\mathbb {S}}^{N-1})$ such that $\mu ^{l_j}\overset{\star }{\rightharpoonup }\mu$ in ${\mathscr {M}}({\mathbb {S}}^{N-1})$ as $j\rightarrow \infty$ and $\mu ({\mathbb {S}}^{N-1})\le M+1$.

Combining Steps 1, 2 and 3 above, we infer the validity of (i), (ii) and (iv), concluding the proof of the first part of the statement of Lemma 2.9.

*Step 4*. Let us now additionally assume that $(\rho _t)_{t\in J}$ has maximal rank as in Definition 2.8. Thus, there exist $\tau \in I$ and a basis $v_1,\dots , v_N\in {\mathbb {S}}^{N-1}$ of $\mathbb {R}^N$ such that ${\mathcal {C}}_\tau (v_i)\cap {\mathcal {C}}_\tau (v_j)=\left\{ 0\right\}$ for every $i,j\in \left\{ 1,\dots ,N\right\}$ such that $i\ne j$, and

2.7 $$\begin{aligned} m{:}{=}\inf _{\delta>0} \, \min _{i\in \left\{ 1,\dots ,N\right\} } \, \liminf _{t\in J,\,t\rightarrow 0^+} \int _{B_\delta \,\cap \,{\mathcal {C}}_\tau (v_i)}\rho _t(z)\,\textrm{d}z>0. \end{aligned}$$

Therefore, we can apply Lemma 2.10, so we let $c_0>0$ be given by Lemma 2.10. Let us now fix $v\in {\mathbb {S}}^{N-1}$. Again by Lemma 2.10, there exists $i_0\in \left\{ 1,\dots ,N\right\}$ such that $|v\cdot \sigma |\ge c_0$ for every $\sigma \in {\mathcal {C}}_\tau (v_{i_0})\cap {\mathbb {S}}^{N-1}$. Now let $(\delta _{l_j})_{j\in \mathbb {N}}$ and $(t_k')_{k\in \mathbb {N}}$ be the sequences defined in Step 3. Recalling (2.2) and setting $\mu _k^j{:}{=}\mu _{t_k'}^{\delta _{l_j}}$ for every $k,j\in \mathbb {N}$, we can write

$$\begin{aligned} \begin{aligned} \Theta _{\mu _k^j}(v)&= \int _{{\mathbb {S}}^{N-1}}|v\cdot \sigma |\,\textrm{d}\mu _k^j(\sigma ) \\&\ge \int _{{\mathbb {S}}^{N-1}\,\cap \,{\mathcal {C}}_\tau (v_{i_0})}|v\cdot \sigma |\,\textrm{d}\mu _k^j(\sigma ) \ge c_0\, \mu _j^k \left( {\mathbb {S}}^{N-1} \cap {\mathcal {C}}_\tau (v_{i_0})\right) \\&= c_0 \int _{B_{\delta _{l_j}}\,\cap \,{\mathcal {C}}_\tau (v_{i_0})} \rho _{t_k'}(z)\,\textrm{d}z \end{aligned} \end{aligned}$$

for every $k,j\in \mathbb {N}$. On the one hand, recalling (2.7), we can estimate

$$\begin{aligned} \begin{aligned} \liminf _{k\rightarrow \infty } \int _{B_{\delta _{l_j}}\,\cap \,{\mathcal {C}}_\tau (v_{i_0})} \rho _{t_k'}(z) \,\textrm{d}z&\ge \liminf _{t\in J,\,t\rightarrow 0^+} \int _{B_{\delta _{l_j}}\,\cap \,{\mathcal {C}}_\tau (v_{i_0})} \rho _t(z)\,\textrm{d}z \\&\ge \min _{i\in \left\{ 1,\dots ,N\right\} } \, \liminf _{t\in J,\,t\rightarrow 0^+} \int _{B_{\delta _{l_j}}\,\cap \,{\mathcal {C}}_\tau (v_i)} \rho _t(z)\,\textrm{d}z \\&\ge \inf _{\delta >0} \, \min _{i\in \left\{ 1,\dots ,N\right\} } \, \liminf _{t\in J,\,t\rightarrow 0^+} \int _{B_\delta \,\cap \,{\mathcal {C}}_\tau (v_i)} \rho _t(z)\,\textrm{d}z =m, \end{aligned} \end{aligned}$$

while, on the other hand, by Step 3, we have

$$\begin{aligned} \liminf _{k\rightarrow \infty } \Theta _{\mu _k^j}(v) = \lim _{k\rightarrow \infty } \int _{{\mathbb {S}}^{N-1}}|v\cdot \sigma |\,\textrm{d}\mu _k^j(\sigma ) = \int _{{\mathbb {S}}^{N-1}}|v\cdot \sigma |\,\textrm{d}\mu ^{l_j}(\sigma ) = \Theta _{\mu ^{l_j}}(v) \end{aligned}$$

for every $j\in \mathbb {N}$. We hence get that $\Theta _{\mu ^{l_j}}(v)\ge c_0\,m$ for every $v\in {\mathbb {S}}^{N-1}$ and $j\in \mathbb {N}$. Again by Step 3, passing to the limit as $j\rightarrow \infty$, we deduce that $\Theta _\mu (v)\ge c_0\,m$ for every $v\in {\mathbb {S}}^{N-1}$; that is, $\inf _{{\mathbb {S}}^{N-1}}\Theta _\mu >0$. The conclusion hence follows from Lemma 2.1. $\square$

### The Functionals ${\mathscr {F}}_{t,p}$

Let $p\in [1,\infty )$. We define ${\mathscr {F}}_{t,p}:L^p(\mathbb {R}^N)\rightarrow [0,\infty ]$ by letting

2.8 $$\begin{aligned} {\mathscr {F}}_{t,p}(u) = \int _{\mathbb {R}^N}\frac{\Vert u(\,\cdot +z)-u\Vert _{L^p}^p}{|z|^p}\,\rho _t(z)\,\textrm{d}z \end{aligned}$$

for $u\in L^p(\mathbb {R}^N)$ and $t\in I$. The following result improves and generalizes [34, Prop. 4.1].

#### Lemma 2.11

Let $J\subset I$ be such that $0\in {\overline{J}}$. If there exists $C>0$ such that

2.9 $$\begin{aligned} \limsup _{t\in J,\,t\rightarrow 0^+} {\mathscr {F}}_{t,p}(u) \le C\Vert Du\Vert _{L^p}^p \end{aligned}$$

for every $u\in \operatorname {Lip}_c(\mathbb {R}^N)$, then any of the properties in Lemma 2.6 holds.

#### Proof

Let $R>0$ and define $u_R\in \operatorname {Lip}_c(\mathbb {R}^N)$ by letting

$$\begin{aligned} u_R(x) = {\left\{ \begin{array}{ll} 1 &  \text {for}\ x\in B_{R/4}, \\ 2-\frac{4}{R}\,|x| &  \text {for}\ x\in B_{R/2}\setminus B_{R/4}, \\ 0 &  \text {for}\ x\in B_{R/2}^c. \end{array}\right. } \end{aligned}$$

Since $\operatorname {supp} u_R\subset B_{R/2}$, we have that

$$\begin{aligned} \Vert u_R(\,\cdot +z)-u_R\Vert _{L^p}^p=2\Vert u_R\Vert _{L^p}^p \ge 2|B_{R/4}| \end{aligned}$$

for every $z\in B_R^c$, from which we deduce that

2.10 $$\begin{aligned} {\mathscr {F}}_{t,p}(u_R) \ge \int _{B_R^c}\frac{\Vert u_R(\,\cdot +z)-u_R\Vert _{L^p}^p}{|z|^p}\,\rho _{t}(z)\,\textrm{d}z \ge 2|B_{R/4}| \int _{B_R^c} \frac{\rho _t(z)}{|z|^p}\,\textrm{d}z.\nonumber \\ \end{aligned}$$

On the other hand, we can estimate

2.11 $$\begin{aligned} \Vert Du_R\Vert _{L^p}^p = \int _{B_{R/2}\setminus B_{R/4}}|Du_R(x)|^p\,\textrm{d}x \le \left( \frac{4}{R}\right) ^p\,|B_{R/2}|. \end{aligned}$$

By combining (2.9) with (2.10) and (2.11), we infer that

2.12 $$\begin{aligned} \limsup _{t\in J,\,t\rightarrow 0^+} \int _{B_R^c} \frac{\rho _t(z)}{|z|^p}\,\textrm{d}z \le C\, \left( \frac{4}{R}\right) ^p\,\frac{|B_{R/2}|}{2|B_{R/4}|} = C\,2^{N-1+2p}\,R^{-p}, \end{aligned}$$

for $R>0$. In view of (iv) in Lemma 2.6, to conclude, we just need to prove that

2.13 $$\begin{aligned} \limsup _{t\in J,\,t\rightarrow 0^+} \int _{B_1}\rho _t(z)\,\textrm{d}z<\infty . \end{aligned}$$

To this end, we define $v\in \operatorname {Lip}_c(\mathbb {R}^N)$ by letting

$$\begin{aligned} v(x) = {\left\{ \begin{array}{ll} e^{|x|^2} &  \text {for}\ x\in B_2, \\ e^4(3-|x|) &  \text {for}\ x\in B_3\setminus B_2, \\ 0 &  \text {for}\ x\in B_3^c. \end{array}\right. } \end{aligned}$$

Since *v* is radially symmetric, a change of variable yields

$$\begin{aligned} \Vert v(\,\cdot +z)-v\Vert _{L^p}^p = \Vert v(\,\cdot +|z|\mathrm e_1)-v\Vert _{L^p}^p \end{aligned}$$

for every $z\in \mathbb {R}^N$. Therefore, letting $D=\left\{ x\in B_1:x_1\ge \frac{1}{2}\right\} \subset B_1$, we can estimate

$$\begin{aligned} \begin{aligned} \Vert v(\,\cdot +z)-v\Vert _{L^p}^p&\ge \int _D\left| e^{|x+|z|\mathrm e_1|^2}-e^{|x|^2}\right| ^p\,\textrm{d}x = \int _D e^{p|x|^2}\left| e^{|z|^2+2|z|x_1}-1\right| ^p\,\textrm{d}x \\&\ge \int _D \left( e^{|z|}-1\right) ^p\,\textrm{d}x \ge |D|\,|z|^p \end{aligned} \end{aligned}$$

for every $z\in B_1$, from which we deduce that

2.14 $$\begin{aligned} {\mathscr {F}}_{t,p}(v) \ge \int _{B_1} \frac{\Vert v(\,\cdot +z)-v\Vert _{L^p}^p}{|z|^p}\,\rho _{t}(z)\,\textrm{d}z \ge |D| \int _{B_1}\rho _{t}(z)\,\textrm{d}z. \end{aligned}$$

By combining (2.14) with (2.9), we infer (2.13) and thus conclude the proof. $\square$

### The Functional ${\mathscr {G}}^{\mu ,\nu }_p$

Given two measures $\mu \in {\mathscr {M}}^+({\mathbb {S}}^{N-1})$ and $\nu \in {\mathscr {M}}^+(\mathbb {R}^N)$, we define ${\mathscr {G}}^{\mu ,\nu }_p: L^p(\mathbb {R}^N)\rightarrow [0,\infty )$ by letting

2.15 $$\begin{aligned} {\mathscr {G}}^{\mu ,\nu }_p(u) = {\left\{ \begin{array}{ll} \displaystyle \int _{{\mathbb {S}}^{N-1}} \Vert \sigma \cdot Du\Vert _{L^p}^p\,\textrm{d}\mu (\sigma ) + \int _{\mathbb {R}^N\setminus \left\{ 0\right\} } \frac{\Vert u(\cdot +z)-u\Vert _{L^p}^p}{|z|^p}\,\textrm{d}\nu (z) &  \text {for}\ u\in {\mathcal {S}^{p}}(\mathbb {R}^N), \\ \infty &  \text {otherwise}. \end{array}\right. }\nonumber \\ \end{aligned}$$

Here, as in (2.3), for every $\sigma \in {\mathbb {S}}^{N-1}$ and $u\in {\mathcal {S}^{p}}(\mathbb {R}^N)$, we have set

$$\begin{aligned} \Vert \sigma \cdot Du\Vert _{L^p}^p = {\left\{ \begin{array}{ll} \displaystyle \int _{\mathbb {R}^N}|\sigma \cdot Du(x)|^p\,\textrm{d}x &  \text {for}\ p>1, \\ |\sigma \cdot Du|(\mathbb {R}^N) &  \text {for}\ p=1. \end{array}\right. } \end{aligned}$$

We observe that, for each fixed $\sigma \in {\mathbb {S}}^{N-1}$, the energy $u\mapsto \Vert \sigma \cdot Du\Vert _{L^p}$ is lower semicontinuous on ${\mathcal {S}^{p}}(\mathbb {R}^N)$ with respect to the $L^p$ convergence of functions.

We collect the basic properties of ${\mathscr {G}}_p^{\mu ,\nu }$ in the following result, whose proof is omitted.

#### Lemma 2.12

Let $p\in [1,\infty )$, $\mu \in {\mathscr {M}}^+({\mathbb {S}}^{N-1})$ and $\nu \in {\mathscr {M}}^+(\mathbb {R}^N)$. The functional ${\mathscr {G}}_p^{\mu ,\nu }$ satisfies the bound

2.16 $$\begin{aligned} {\mathscr {G}}^{\mu ,\nu }_p(u) \le \Vert Du\Vert _{L^p}^p\left( \mu ({\mathbb {S}}^{N-1})+\nu (\mathbb {R}^N\setminus \left\{ 0\right\} )\right) \end{aligned}$$

for every $u\in {\mathcal {S}^{p}}(\mathbb {R}^N)$. Moreover, if $(u_k)_{k\in \mathbb {N}}\subset {\mathcal {S}^{p}}(\mathbb {R}^N)$ is such that $u_k\rightarrow u$ in $L^p(\mathbb {R}^N)$ as $k\rightarrow \infty$ for some $u\in {\mathcal {S}^{p}}(\mathbb {R}^N)$, then

2.17 $$\begin{aligned} \liminf _{k\rightarrow \infty } {\mathscr {G}}^{\mu ,\nu }_p(u_k) \ge {\mathscr {G}}^{\mu ,\nu }_p(u). \end{aligned}$$

The following results build upon the ideas contained in the proof of [34, Th. 1.1].

#### Lemma 2.13

If $\lambda ,\mu \in {\mathscr {M}}^+({\mathbb {S}}^{N-1})$, $\nu \in {\mathscr {M}}^+(\mathbb {R}^N)$ and $\alpha \in [0,\infty )$ are such that ${\mathscr {G}}^{\mu ,\nu }_p(u)={\mathscr {G}}^{\lambda ,\alpha \delta _0}_p(u)$ for every $u\in \operatorname {Lip}_c(\mathbb {R}^N)$, then $\nu =\beta \delta _0$ for some $\beta \in [0,\infty )$.

#### Proof

We let $u\in \operatorname {Lip}_c(\mathbb {R}^N)$ and we define $u_\varepsilon =\varepsilon ^{1-\frac{N}{p}}\,u(\cdot /\varepsilon )$ for every $\varepsilon >0$. We note that $u_\varepsilon \in \operatorname {Lip}_c(\mathbb {R}^N)$ for every $\varepsilon >0$. Moreover, we have that

$$\begin{aligned} \Vert \sigma \cdot Du_\varepsilon \Vert _{L^p}^p = \left\| \varepsilon ^{-\frac{N}{p}}\,\sigma \cdot Du(\cdot /\varepsilon )\right\| _{L^p}^p = \Vert \sigma \cdot Du\Vert _{L^p}^p \end{aligned}$$

for every $\sigma \in {\mathbb {S}}^{N-1}$ and, similarly,

$$\begin{aligned} \Vert u_\varepsilon (\,\cdot +z)-u_\varepsilon \Vert _{L^p}^p = \varepsilon ^N \left\| \varepsilon ^{1-\frac{N}{p}}\left( u\left( \,\cdot +\tfrac{z}{\varepsilon }\right) -u\right) \right\| _{L^p}^p = \varepsilon ^p\left\| u\left( \,\cdot +\tfrac{z}{\varepsilon }\right) -u\right\| _{L^p}^p \end{aligned}$$

for every $z\in \mathbb {R}^N$. Thus, the equality ${\mathscr {G}}^{\mu ,\nu }_p(u_\varepsilon )={\mathscr {G}}^{\lambda ,\alpha \delta _0}_p(u_\varepsilon )$ equivalently rewrites as

2.18 $$\begin{aligned}  &   \int _{{\mathbb {S}}^{N-1}}\Vert \sigma \cdot Du\Vert _{L^p}^p\,\textrm{d}\mu (\sigma ) + \varepsilon ^p\int _{\mathbb {R}^N\setminus \left\{ 0\right\} }\frac{\left\| u\left( \,\cdot +\tfrac{z}{\varepsilon }\right) -u\right\| _{L^p}^p}{|z|^p} \,\textrm{d}\nu (z)\nonumber \\  &   \quad =\int _{{\mathbb {S}}^{N-1}}\Vert \sigma \cdot Du\Vert _{L^p}^p\,\textrm{d}\lambda (\sigma ) \end{aligned}$$

for every $\varepsilon >0$. We claim that

2.19 $$\begin{aligned} \lim _{\varepsilon \rightarrow 0^+} \varepsilon ^p\int _{\mathbb {R}^N\setminus \left\{ 0\right\} } \frac{\left\| u\left( \,\cdot +\tfrac{z}{\varepsilon }\right) -u\right\| _{L^p}^p}{|z|^p} \,\textrm{d}\nu (z) =0. \end{aligned}$$

Indeed, by Lemma 2.2(i), we can estimate that

2.20 $$\begin{aligned} \varepsilon ^p\,\frac{\left\| u\left( \,\cdot +\tfrac{z}{\varepsilon }\right) -u\right\| _{L^p}^p}{|z|^p} \le \Vert Du\Vert _{L^p}^p \end{aligned}$$

for every $z\in \mathbb {R}^N\setminus \left\{ 0\right\}$ and $\varepsilon >0$. Moreover, we have that

2.21 $$\begin{aligned} \limsup _{\varepsilon \rightarrow 0^+} \varepsilon ^p\,\frac{\left\| u\left( \,\cdot +\tfrac{z}{\varepsilon }\right) -u\right\| _{L^p}^p}{|z|^p} \le \limsup _{\varepsilon \rightarrow 0^+} \varepsilon ^p \, \frac{2^p\Vert u\Vert _{L^p}^p}{|z|^p} =0 \end{aligned}$$

for every $z\in \mathbb {R}^N\setminus \left\{ 0\right\}$. Owing to (2.20) and (2.21), the claimed (2.19) follows by the Dominated Convergence Theorem applied with respect to $\nu$. Therefore, passing to the limit in (2.18) as $\varepsilon \rightarrow 0^+$ and exploiting (2.19), we conclude that

$$\begin{aligned} \int _{{\mathbb {S}}^{N-1}}\Vert \sigma \cdot Du\Vert _{L^p}^p\,\textrm{d}\mu (\sigma ) = \int _{{\mathbb {S}}^{N-1}}\Vert \sigma \cdot Du\Vert _{L^p}^p\,\textrm{d}\lambda (\sigma ) \end{aligned}$$

whenever $u\in \operatorname {Lip}_c(\mathbb {R}^N)$. By our initial assumption, this means that

$$\begin{aligned} \int _{\mathbb {R}^N\setminus \left\{ 0\right\} }\frac{\Vert u(\,\cdot +z)-u\Vert _{L^p}^p}{|z|^p} \,\textrm{d}\nu (z) = 0 \end{aligned}$$

for every $u\in \operatorname {Lip}_c(\mathbb {R}^N)$. In particular, if $u\in \operatorname {Lip}_c(\mathbb {R}^N)$ is such that $\operatorname {supp} u\subset B_{\varepsilon /2}$ for some $\varepsilon >0$, then

$$\begin{aligned} 0 \ge \int _{B_\varepsilon ^c}\frac{\Vert u(\,\cdot +z)-u\Vert _{L^p}^p}{|z|^p} \,\textrm{d}\nu (z) = 2\Vert u\Vert _{L^p}^p \int _{B_\varepsilon ^c} \frac{\,\textrm{d}\nu (z)}{|z|^p}, \end{aligned}$$

from which we deduce that $\nu (B_\varepsilon ^c)=0$ whenever $\varepsilon >0$. Thus $\nu (\mathbb {R}^N\setminus \left\{ 0\right\} )=0$, from which we get that $\nu =\beta \delta _0$ for some $\beta \in [0,\infty )$, concluding the proof. $\square$

## Proof of Theorems 1.1 and 1.2

Throughout this section, we let $p\in [1,\infty )$ and $(\rho _t)_{t\in I}\subset L^1_\textrm{loc}(\mathbb {R}^N)$ be such that $\rho _t\ge 0$ for every $t\in I$. To prove Theorems 1.1 and 1.2, we need the following preliminary result, which improves and generalizes [34, Th. 1.2].

### Theorem 3.1

Let $J\subset I$ be such that $0\in {\overline{J}}$. If any of the properties in Lemma 2.6 hold, then there exists an infinitesimal sequence $(t_k)_{k\in \mathbb {N}}\subset J$ and two measures $\mu \in {\mathscr {M}}^+({\mathbb {S}}^{N-1})$ and $\nu \in {\mathscr {M}}^+(\mathbb {R}^N)$ such that the following hold:

i $$\rho _{t_k}\,{\mathscr {L}}^N\overset{\star }{\rightharpoonup }\nu $$ in $${\mathscr {M}}_\textrm{loc}(\mathbb {R}^N)$$ as $$k\rightarrow \infty $$;
ii if $u\in {\mathcal {S}^{p}}(\mathbb {R}^N)$, then $\displaystyle \limsup _{k\rightarrow \infty } {\mathscr {F}}_{t_k,p}(u) \le {\mathscr {G}}^{\mu ,\nu }_p(u)$;
iii if $(u_k)_{k\in \mathbb {N}}\subset L^p(\mathbb {R}^N)$ is such that $u_k\rightarrow u$ in $L^p(\mathbb {R}^N)$ as $k\rightarrow \infty$ for some $u\in {\mathcal {S}^{p}}(\mathbb {R}^N)$, then $\displaystyle \liminf _{k\rightarrow \infty } {\mathscr {F}}_{t_k,p}(u_k) \ge {\mathscr {G}}^{\mu ,\nu }_p(u)$.

As a consequence, $({\mathscr {F}}_{t_k,p})_{k\in \mathbb {N}}$ converges to ${\mathscr {G}}_p^{\mu ,\nu }$ on ${\mathcal {S}^{p}}(\mathbb {R}^N)$ as $k\rightarrow \infty$ pointwise and in the $\Gamma$-sense with respect to the $L^p$ topology. In addition, if $(\rho _t)_{t\in J}$ has maximal rank, then the family $({\mathscr {F}}_{t_k,p})_{k\in \mathbb {N}}$ is coercive on ${\mathcal {S}^{p}}(\mathbb {R}^N)$.

### Proof

For every measurable set $A\subset \mathbb {R}^N$, $u\in {\mathcal {S}^{p}}(\mathbb {R}^N)$ and $t\in I$, we set

$$\begin{aligned} {\mathscr {F}}_{t,p}(u;A) = \int _A\frac{\Vert u(\,\cdot +z)-u\Vert _{L^p}^p}{|z|^p}\,\rho _t(z)\,\textrm{d}z. \end{aligned}$$

Note that ${\mathscr {F}}_{t,p}(u;\mathbb {R}^N)={\mathscr {F}}_{t,p}(u)$ for every $t\in I$ and $u\in {\mathcal {S}^{p}}(\mathbb {R}^N)$. Owing to Lemma 2.6, there exists $M>0$ such that

3.1 $$\begin{aligned} \limsup _{t\rightarrow 0^+} \int _{B_R}\rho _t(z)\,\textrm{d}z+ \limsup _{t\rightarrow 0^+} R^p\int _{B_R^c}\frac{\rho _t(z)}{|z|^p}\,\textrm{d}z \le M \end{aligned}$$

for every $R>0$. By (3.1), we can apply Lemma 2.9 and find a countable set $I_0\subset I$, two infinitesimal sequences $(t_k)_{k\in \mathbb {N}}\subset J$ and $(\delta _l)_{l\in \mathbb {N}}\subset I\setminus I_0$, and two measures $\mu \in {\mathscr {M}}^+({\mathbb {S}}^{N-1})$ and $\nu \in {\mathscr {M}}^+(\mathbb {R}^N)$ satisfying the statements (i), (ii) and (iv) of Lemma 2.9. In particular, this immediately yields (i). We shall prove (ii) and (iii) separately. In what follows, we let $(\mu _k^l)_{k,l\in \mathbb {N}}\subset {\mathscr {M}}^+({\mathbb {S}}^{N-1})$, $\mu _k^l{:}{=}\mu _{t_k}^{\delta _l}$ for every $k,l\in \mathbb {N}$, and $(\mu ^l)_{l\in \mathbb {N}}\subset {\mathscr {M}}^+({\mathbb {S}}^{N-1})$ be given as in statement (iv) of Lemma 2.9.

*Proof of* (ii). We begin by observing that

3.2 $$\begin{aligned} {\mathscr {F}}_{t,p}(u;\mathbb {R}^N) = {\mathscr {F}}_{t,p}(u;B_\delta ) + {\mathscr {F}}_{t,p}(u;A_\delta ) + {\mathscr {F}}_{t,p}(u;B_{1/\delta }^c) \end{aligned}$$

for every $t,\delta \in I$, where $A_\delta =\left\{ x\in \mathbb {R}^N : \delta<|x|<\frac{1}{\delta }\right\}$ as in Lemma 2.9. We now deal with each piece on the right-hand side of (3.2) separately. By Lemma 2.2, we can estimate

$$\begin{aligned} {\mathscr {F}}_{t_k,p}(u;B_{\delta _l}) \le \int _{B_{\delta _l}}\left\| \tfrac{z}{|z|}\cdot Du\right\| _{L^p}^p\,\textrm{d}\nu _k(z) = \int _{{\mathbb {S}}^{N-1}} \Vert \sigma \cdot Du\Vert _{L^p}^p\,\textrm{d}\mu _k^l(\sigma ) \end{aligned}$$

for every $u\in {\mathcal {S}^{p}}(\mathbb {R}^N)$ and $k,l\in \mathbb {N}$ (recall the definition in (2.4) in Lemma 2.9). Since $\sigma \mapsto \Vert \sigma \cdot Du\Vert _{L^p}^p\in C({\mathbb {S}}^{N-1})$ for every $u\in {\mathcal {S}^{p}}(\mathbb {R}^N)$, by Lemma 2.9(iv) we get that

3.3 $$\begin{aligned} \begin{aligned} \lim _{l\rightarrow \infty }&\limsup _{k\rightarrow \infty } {\mathscr {F}}_{t_k,p}(u;B_{\delta _l}) \le \lim _{l\rightarrow \infty } \lim _{k\rightarrow \infty } \int _{{\mathbb {S}}^{N-1}} \Vert \sigma \cdot Du\Vert _{L^p}^p\,\textrm{d}\mu _k^l(\sigma ) \\&= \lim _{l\rightarrow \infty } \int _{{\mathbb {S}}^{N-1}} \Vert \sigma \cdot Du\Vert _{L^p}^p\,\textrm{d}\mu ^l(\sigma ) = \int _{{\mathbb {S}}^{N-1}} \Vert \sigma \cdot Du\Vert _{L^p}^p\,\textrm{d}\mu (\sigma ). \end{aligned} \end{aligned}$$

Moreover, since

3.4 $$\begin{aligned} z\mapsto f_u(z)=\frac{\Vert u(\,\cdot +z)-u\Vert _{L^p}^p}{|z|^p}\in C(\mathbb {R}^N\setminus \left\{ 0\right\} ) \end{aligned}$$

for every $u\in {\mathcal {S}^{p}}(\mathbb {R}^N)$, by Lemma 2.9(ii) and by the Monotone Convergence Theorem, we also have that

3.5 $$\begin{aligned} \begin{aligned} \lim _{l\rightarrow \infty } \lim _{k\rightarrow \infty } {\mathscr {F}}_{t_k,p}(u;A_l)&= \lim _{l\rightarrow \infty } \lim _{k\rightarrow \infty } \int _{A_l} f_u\,\textrm{d}\nu _k = \lim _{l\rightarrow \infty } \int _{A_l} f_u\,\textrm{d}\nu = \int _{\mathbb {R}^N\setminus \left\{ 0\right\} } f_u\,\textrm{d}\nu \\&= \int _{\mathbb {R}^N\setminus \left\{ 0\right\} } \frac{\Vert u(\cdot +z)-u\Vert _{L^p}^p}{|z|^p}\,\textrm{d}\nu (z) \end{aligned} \end{aligned}$$

for every $u\in {\mathcal {S}^{p}}(\mathbb {R}^N)$, where $A_l=A_{\delta _l}$ for every $l\in \mathbb {N}$ as in Lemma 2.9(ii). Finally, by exploiting (3.1), we can estimate

3.6 $$\begin{aligned} \begin{aligned} \lim _{l\rightarrow \infty } \limsup _{k\rightarrow \infty } {\mathscr {F}}_{t_k,p}(u;B_{1/\delta _l}^c)&\le 2^p\,\Vert u\Vert _{L^p}^p \lim _{l\rightarrow \infty } \limsup _{k\rightarrow \infty } \int _{B_{1/\delta _l}^c} \frac{\rho _{t_k}(z)}{|z|^p}\,\textrm{d}z \\&\le 2^p\,\Vert u\Vert _{L^p}^p\,M \lim _{l\rightarrow \infty } \delta ^p_l=0 \end{aligned} \end{aligned}$$

for every $u\in {\mathcal {S}^{p}}(\mathbb {R}^N)$. By combining (3.3), (3.5) and (3.6) with (3.2), we get (ii).

*Proof of* (iii). Let $(u_k)_{k\in \mathbb {N}}\subset L^p(\mathbb {R}^N)$ and $u\in {\mathcal {S}^{p}}(\mathbb {R}^N)$ be such that $u_k\rightarrow u$ in $L^p(\mathbb {R}^N)$ as $k\rightarrow \infty$. Let $(\eta _j)_{j\in \mathbb {N}}\subset C^\infty _c(\mathbb {R}^N)$ be a sequence of mollifiers and set $u_k^j=u_k*\eta _j$ and $u^j=u*\eta _j$ for every $k,j\in \mathbb {N}$. We observe that $u_k^j,u^j\in {\mathcal {S}^{p}}(\mathbb {R}^N)\cap C^\infty (\mathbb {R}^N)$ with $D^2 u_k^j,D^2u^j\in L^p(\mathbb {R}^N)$ for every $k,j\in \mathbb {N}$. Moreover, by Young’s inequality, we have that

3.7 $$\begin{aligned} u_k^j\rightarrow u^j, \ Du_k^j\rightarrow Du^j \ \text {and} \ D^2u_k^j\rightarrow D^2u^j \ \text {in } L^p(\mathbb {R}^N) \text { as } k\rightarrow \infty \end{aligned}$$

for every $j\in \mathbb {N}$. In addition, again by Young’s inequality,

$$\begin{aligned} \Vert u_k^j(\,\cdot +z)-u_k^j\Vert _{L^p} = \Vert (u_k(\,\cdot +z)-u_k)*\eta _j\Vert _{L^p} \le \Vert u_k(\,\cdot +z)-u_k\Vert _{L^p} \end{aligned}$$

for every $k,j\in \mathbb {N}$ and $z\in \mathbb {R}^N$, from which we get that ${\mathscr {F}}_{t,p}(u_k^j;A)\le {\mathscr {F}}_{t,p}(u_k;A)$ for every measurable set $A\subset \mathbb {R}^N$ and every $k,j\in \mathbb {N}$. Consequently, we have that

$$\begin{aligned} \liminf _{k\rightarrow \infty } {\mathscr {F}}_{t_k,p}(u_k) \ge \liminf _{k\rightarrow \infty } {\mathscr {F}}_{t_k,p}(u_k^j) \end{aligned}$$

for every $j\in \mathbb {N}$. We now claim that

3.8 $$\begin{aligned} \lim _{k\rightarrow \infty } {\mathscr {F}}_{t_k,p}(u_k^j) = {\mathscr {G}}^{\mu ,\nu }_p(u^j) \end{aligned}$$

for every $j\in \mathbb {N}$. To prove (3.8), we argue as in the proof of (ii) by again relying on (3.2). Indeed, as in (3.6), observing that $\Vert u_k^j\Vert _{L^p}\le \Vert u_k\Vert _{L^p}$ for every $k,j\in \mathbb {N}$, we have that

$$\begin{aligned} \lim _{l\rightarrow \infty } \lim _{k\rightarrow \infty } {\mathscr {F}}_{t_k,p}(u_k^j;B_{1/\delta _l}^c) = 2^p\,\Vert u^j\Vert _{L^p}^p\,M \lim _{l\rightarrow \infty } \delta ^p_l=0 \end{aligned}$$

for every $j\in \mathbb {N}$. Moreover, as in (3.5), since $f_{u_k^j}\rightarrow f_{u^j}$ locally uniformly on $\mathbb {R}^N\setminus \left\{ 0\right\}$ as $k\rightarrow \infty$ for every $j\in \mathbb {N}$ owing to (3.7) (for the notation, recall (3.4)), we also have that

$$\begin{aligned} \begin{aligned} \lim _{l\rightarrow \infty } \lim _{k\rightarrow \infty } {\mathscr {F}}_{t_k,p}(u_k^j;A_l)&= \lim _{l\rightarrow \infty } \lim _{k\rightarrow \infty } \int _{A_l}f_{u_k^j}\,\textrm{d}\nu _k = \lim _{l\rightarrow \infty } \int _{A_l}f_{u^j}\,\textrm{d}\nu = \int _{\mathbb {R}^N\setminus \left\{ 0\right\} }f_{u^j}\,\textrm{d}\nu \\&= \int _{\mathbb {R}^N\setminus \left\{ 0\right\} } \frac{\Vert u^j(\cdot +z)-u^j\Vert _{L^p}^p}{|z|^p}\,\textrm{d}\nu (z) \end{aligned} \end{aligned}$$

for every $j\in \mathbb {N}$. In addition, as in (3.3), since the functions $\sigma \mapsto \Vert \sigma \cdot Du_k^j\Vert _{L^p}^p$ converge uniformly on ${\mathbb {S}}^{N-1}$ to $\sigma \mapsto \Vert \sigma \cdot Du^j\Vert _{L^p}^p$ as $k\rightarrow \infty$ for every $j\in \mathbb {N}$ owing to (3.7), we get

$$\begin{aligned} \begin{aligned} \lim _{l\rightarrow \infty } \lim _{k\rightarrow \infty } \int _{B_{\delta _l}}&\left\| \tfrac{z}{|z|}\cdot Du_k^j\right\| _{L^p}^p\,\textrm{d}\nu _k(z) = \lim _{l\rightarrow \infty } \lim _{k\rightarrow \infty } \int _{{\mathbb {S}}^{N-1}} \Vert \sigma \cdot Du_k^j\Vert _{L^p}^p\,\textrm{d}\mu _k^l(\sigma ) \\&= \lim _{l\rightarrow \infty } \int _{{\mathbb {S}}^{N-1}} \Vert \sigma \cdot Du^j\Vert _{L^p}^p\,\textrm{d}\mu ^l(\sigma ) = \int _{{\mathbb {S}}^{N-1}} \Vert \sigma \cdot Du^j\Vert _{L^p}^p\,\textrm{d}\mu (\sigma ) \end{aligned} \end{aligned}$$

for every $j\in \mathbb {N}$ (recall the definition in (2.4) in Lemma 2.9). Therefore, in view of (3.2), the claim in (3.8) follows if we prove that

$$\begin{aligned} \lim _{l\rightarrow \infty } \lim _{k\rightarrow \infty } {\mathscr {F}}_{t_k,p}(u_k^j;B_{\delta _l}) = \int _{{\mathbb {S}}^{N-1}} \Vert \sigma \cdot Du^j\Vert _{L^p}^p\,\textrm{d}\mu (\sigma ); \end{aligned}$$

that is, equivalently, we just have to show that

3.9 $$\begin{aligned} \lim _{l\rightarrow \infty } \lim _{k\rightarrow \infty } \left| {\mathscr {F}}_{t_k,p}(u_k^j;B_{\delta _l}) - \int _{B_{\delta _l}}\left\| \tfrac{z}{|z|}\cdot Du_k^j\right\| _{L^p}^p\,\textrm{d}\nu _k(z) \right| = 0 \end{aligned}$$

for every $j\in \mathbb {N}$. To this end, we start by noticing that, by Lemma 2.2(ii),

$$\begin{aligned} \left| \frac{\Vert u_k^j(\,\cdot +z)-u_k^j\Vert _{L^p}}{|z|} - \left\| \tfrac{z}{|z|}\cdot Du_k^j\right\| _{L^p} \right| \le \frac{|z|}{2}\,\Vert D^2u_k^j\Vert _{L^p} \end{aligned}$$

for every $z\in \mathbb {R}^N\setminus \left\{ 0\right\}$. Hence, since $|a^p-b^p|\le p\max \left\{ a,b\right\} ^{p-1}|a-b|$, for all $a,b\ge 0$, and

$$\begin{aligned} \max \left\{ \frac{\Vert u_k^j(\,\cdot +z)-u_k^j\Vert _{L^p}}{|z|}, \left\| \tfrac{z}{|z|}\cdot Du_k^j\right\| _{L^p} \right\} \le \Vert Du_k^j\Vert _{L^p} \end{aligned}$$

for every $k,j\in \mathbb {N}$ and $z\in \mathbb {R}^N\setminus \left\{ 0\right\}$ by Lemma 2.2, we can estimate

$$\begin{aligned} \begin{aligned} \left| \frac{\Vert u_k^j(\,\cdot +z)-u_k^j\Vert _{L^p}^p}{|z|^p} - \left\| \tfrac{z}{|z|}\cdot Du_k^j\right\| _{L^p}^p \right|&\le p\, \Vert Du_k^j\Vert _{L^p}^{p-1} \, \left| \frac{\Vert u_k^j(\,\cdot +z)-u_k^j\Vert _{L^p}}{|z|} - \left\| \tfrac{z}{|z|}\cdot Du_k^j\right\| _{L^p} \right| \\&\le \frac{p|z|}{2} \, \Vert Du_k^j\Vert _{L^p}^{p-1} \, \Vert D^2u_k^j\Vert _{L^p}, \end{aligned} \end{aligned}$$

for every $i,j\in \mathbb {N}$ and $z\in \mathbb {R}^N\setminus \left\{ 0\right\}$. Consequently, we get that

$$\begin{aligned} \left| {\mathscr {F}}_{t_k,p}(u_k^j;B_{\delta _l}) - \!\!\int _{B_{\delta _l}}\left\| \tfrac{z}{|z|}\cdot Du_k^j\right\| _{L^p}^p\,\textrm{d}\nu _k(z) \right| \! \le \frac{p}{2} \, \Vert Du_k^j\Vert _{L^p}^{p-1} \, \Vert D^2u_k^j\Vert _{L^p} \, \delta _l\,\nu _k(B_{\delta _l}) \end{aligned}$$

for every $k,j,l\in \mathbb {N}$, from which, owing to (3.1) and (3.7), the claimed (3.9) follows. We thus completed the proof of (3.8) and so, by the lower semicontinuity of ${\mathscr {G}}^{\mu ,\nu }_p$ with respect to the $L^p$ convergence of functions in ${\mathcal {S}^{p}}(\mathbb {R}^N)$ (recall (2.17)),

$$\begin{aligned} \liminf _{k\rightarrow \infty } {\mathscr {F}}_{t_k,p}(u_k) \ge \liminf _{j\rightarrow \infty } {\mathscr {G}}^{\mu ,\nu }_p(u^j) = {\mathscr {G}}^{\mu ,\nu }_p(u), \end{aligned}$$

concluding the proof of (iii).

With (ii) and (iii) in force, the convergence of $({\mathscr {F}}_{t_k,p})_{k\in \mathbb {N}}$ to ${\mathscr {G}}_p^{\mu ,\nu }$ on ${\mathcal {S}^{p}}(\mathbb {R}^N)$ as $k\rightarrow \infty$ in the pointwise and $\Gamma$-sense with respect to the $L^p$ topology follows by Definition 2.4. We are thus left to prove that, if $(\rho _t)_{t\in J}$ has maximal rank, then $({\mathscr {F}}_{t_k,p})_{k\in \mathbb {N}}$ is coercive. Indeed, let $(u_k)_{k\in \mathbb {N}}\subset L^p(\mathbb {R}^N)$ be such that $u_k\rightarrow u$ in $L^p(\mathbb {R}^N)$ as $k\rightarrow \infty$ for some $u\in L^p(\mathbb {R}^N)$ and $C=\liminf \limits _{k\rightarrow \infty } {\mathscr {F}}_{t_k,p}(u_k)\in [0,\infty )$. Let us define $u_k^j=u_k*\eta _j$ and $u^j=u*\eta _j$ for every $k,j\in \mathbb {N}$ as in the proof of (iii). We observe that $u_k^j,u\in {\mathcal {S}^{p}}(\mathbb {R}^N)$ with ${\mathscr {F}}_{t,p}(u_k^j)\le {\mathscr {F}}_{t,p}(u_k)$ for every $k,j\in \mathbb {N}$ and that $u_k^j\rightarrow u^j$ in $L^p(\mathbb {R}^N)$ as $k\rightarrow \infty$ for every $j\in \mathbb {N}$. Therefore, thanks to (iii), we get that

$$\begin{aligned} C \ge \liminf _{k\rightarrow \infty } {\mathscr {F}}_{t_k,p}(u_k^j) \ge {\mathscr {G}}_p^{\mu ,\nu }(u^j) \end{aligned}$$

for every $j\in \mathbb {N}$. We now note that

$$\begin{aligned} {\mathscr {G}}_p^{\mu ,\nu }(u^j) \ge \int _{{\mathbb {S}}^{N-1}}\Vert \sigma \cdot Du^j\Vert _{L^p}^p\,\textrm{d}\mu (\sigma ) = \int _{\mathbb {R}^N} \int _{{\mathbb {S}}^{N-1}} |\sigma \cdot Du^j(x)|^p \,\textrm{d}\mu (\sigma ) \,\textrm{d}x. \end{aligned}$$

By Jensen’s inequality, and recalling the notation introduced in (2.2), we have that

$$\begin{aligned} \begin{aligned} \int _{{\mathbb {S}}^{N-1}} |\sigma \cdot Du^j(x)|^p \,\textrm{d}\mu (\sigma )&\ge \mu ({\mathbb {S}}^{N-1})^{1-p} \left( \int _{{\mathbb {S}}^{N-1}} |\sigma \cdot Du^j(x)| \,\textrm{d}\mu (\sigma ) \right) ^p \\&= \mu ({\mathbb {S}}^{N-1})^{1-p} \, \Theta _\mu (Du^j(x))^p, \end{aligned} \end{aligned}$$

so that $\sup _{j\in \mathbb {N}}\Vert \Theta _\mu (Du^j)\Vert _{L^p}<\infty$. Now, since $(\rho _t)_{t\in J}$ has maximal rank, by Lemma 2.9 we get that $\operatorname {span}(\operatorname {supp}\mu )=\mathbb {R}^N$ and thus, by Lemma 2.1, we infer that

$$\begin{aligned} \alpha {:}{=}\min _{v\in {\mathbb {S}}^{N-1}}\Theta _\mu (v)\in (0,\infty ). \end{aligned}$$

As a consequence, we deduce that

$$\begin{aligned} \infty > \sup _{j\in \mathbb {N}}\Vert \Theta _\mu (Du^j)\Vert _{L^p} \ge \alpha \sup _{j\in \mathbb {N}}\Vert Du^j\Vert _{L^p}, \end{aligned}$$

proving that $(u^j)_{j\in \mathbb {N}}$ is a bounded sequence in $\mathcal {S}^{p}(\mathbb {R}^N)$. Since $u^j\rightarrow u$ in $L^p(\mathbb {R}^N)$ as $j\rightarrow \infty$, we get that $u\in {\mathcal {S}^{p}}(\mathbb {R}^N)$, concluding the proof. $\square$

### Proof of Theorems 1.1 and 1.2

We begin with the proof of Theorem 1.1, showing the equivalence between (A) and (B) by dealing with the two implications separately.

*Proof of* (A)$\implies$(B). If (A) holds, then, by Theorem 3.1, we find two measures $\mu \in {\mathscr {M}}^+({\mathbb {S}}^{N-1})$ and $\nu \in {\mathscr {M}}^+(\mathbb {R}^N)$ such that $({\mathscr {F}}_{t_k,p})_{k\in \mathbb {N}}$ converges to ${\mathscr {G}}_p^{\mu ,\nu }$ on ${\mathcal {S}^{p}}(\mathbb {R}^N)$ as $k\rightarrow \infty$ pointwise and in the $\Gamma$-sense with respect to the $L^p$ topology. Actually, Theorem 3.1(i) yields that $\nu =\alpha \delta _0$ for some $\alpha \in [0,\infty )$, so that ${\mathscr {G}}_p^{\mu ,\alpha \delta _0}={\mathscr {G}}_p^{\mu ,0}={\mathscr {D}}_p^\mu$ on ${\mathcal {S}^{p}}(\mathbb {R}^N)$, proving (B).

*Proof of* (B)$\implies$(A). If (B) holds, then (2.16) yields that

$$\begin{aligned} \lim _{k\rightarrow \infty } {\mathscr {F}}_{t_k,p}(u) = {\mathscr {D}}_p^\mu (u)= {\mathscr {G}}^{\mu ,0}_p(u) \le \mu ({\mathbb {S}}^{N-1}) \, \Vert Du\Vert _{L^p}^p \end{aligned}$$

for every $u\in {\mathcal {S}^{p}}(\mathbb {R}^N)$. We can thus apply Lemma 2.11 and then Lemma 2.6 to get the first part of (A). This, in turn, allows us to apply Theorem 3.1 and find two measures $\lambda \in {\mathscr {M}}^+({\mathbb {S}}^{N-1})$ and $\nu \in {\mathscr {M}}^+(\mathbb {R}^N)$ such that $\rho _{t_k}\,{\mathscr {L}}^N\overset{\star }{\rightharpoonup }\nu$ in ${\mathscr {M}}_\textrm{loc}(\mathbb {R}^N)$ as $k\rightarrow \infty$ and, moreover, $({\mathscr {F}}_{t_k,p})_{k\in \mathbb {N}}$ converges to ${\mathscr {G}}_p^{\lambda ,\nu }$ on ${\mathcal {S}^{p}}(\mathbb {R}^N)$ as $k\rightarrow \infty$ pointwise and in the $\Gamma$-sense with respect to the $L^p$ topology. Because of (B), this means that ${\mathscr {G}}_p^{\lambda ,\nu }={\mathscr {D}}_p^\mu ={\mathscr {G}}_p^{\mu ,0}$ on ${\mathcal {S}^{p}}(\mathbb {R}^N)$ and thus, by Lemma 2.13, we conclude that $\nu =\alpha \delta _0$ for some $\alpha \in [0,\infty )$, proving the second part of (A).

To prove Theorem 1.2, so further assuming that $(\rho _{t_k})_{k\in \mathbb {N}}$ is of maximal rank as in Definition 2.8, it is enough to observe that, if (A) or (B) holds, then Theorem 3.1 yields that $({\mathscr {F}}_{t_k,p})_{k\in \mathbb {N}}$ is coercive on ${\mathcal {S}^{p}}(\mathbb {R}^N)$. $\square$

### Remark 3.2

Given $m\in \mathbb {N}$ such that $m\le N$, write $\mathbb {R}^N=\mathbb {R}^m\times \mathbb {R}^{N-m}$ and $x=(x',x'')$ accordingly, and let $B_r^m=\left\{ x'\in \mathbb {R}^m: |x'|<r\right\}$ and $B_r^{N-m}=\left\{ x''\in \mathbb {R}^{N-m}: |x''|<r\right\}$ for all $r>0$. The family $(\rho _t^{(m,1)})_{t\in I}\subset L^1(\mathbb {R}^N)$ defined as

$$\begin{aligned} \rho _t^{(m,1)}{:}{=}\frac{\chi _{B_t^m\times B_{t^2}^{N-m}}}{{\mathscr {L}}^m(B_t^m)\cdot {\mathscr {L}}^{N-m}(B_{t^2}^{N-m})}, \quad t\in I, \end{aligned}$$

is such that $\Vert \rho ^{(m,1)}_t\Vert _{L^1}=1$ for all $t\in I$ (and thus $(\rho ^{(m,1)}_t)_{t\in I}$ obviously satisfies (1.4) as $t\rightarrow 0^+$) and $\nu _t^{(m,1)}{:}{=}\rho _t^{(m,1)}{\mathscr {L}}^N\overset{\star }{\rightharpoonup }\delta _0$ in ${\mathscr {M}}_\textrm{loc}(\mathbb {R}^N)$ as $t\rightarrow 0^+$. Therefore, the family $(\rho _t^{(m,1)})_{t\in I}$ satisfies part (A) of Theorem 1.1 along *any* infinitesimal sequence $(t_k)_{k\in \mathbb {N}}\subset I$. By [61, Ex. 3], part (B) of Theorem 1.1 holds withwhere

$$\begin{aligned} {\mathbb {S}}^{m-1,1}{:}{=}\left\{ x\in \mathbb {R}^N: |x'|=1\right\} , \quad c_{m,1}{:}{=}{\mathscr {H}}^{m-1}({\mathbb {S}}^{m-1,1})^{-1}, \end{aligned}$$

along *any* infinitesimal sequence $(t_k)_{k \in \mathbb {N}}$. In particular, the limit Dirichlet energy is

$$\begin{aligned} {\mathscr {D}}^{(m,1)}_p(u) = c_m\int _{{\mathbb {S}}^{m-1,1}}\Vert \sigma \cdot Du\Vert _{L^p}^p\,\textrm{d}{\mathscr {H}}^{m-1}(\sigma ), \quad u\in \mathcal {S}^{p}(\mathbb {R}^N), \end{aligned}$$

for any $p\in [1,\infty )$, see [61, Cor. 2] and the comments below it. *Mutatis mutandis*, also the family $(\rho _t^{(m,2)})_{t\in I}\subset L^1(\mathbb {R}^N)$ defined as

$$\begin{aligned} \rho _t^{(m,2)}{:}{=}\frac{\chi _{B_{t^2}^m\times B_t^{N-m}}}{{\mathscr {L}}^m(B_{t^2}^m)\cdot {\mathscr {L}}^{N-m}(B_t^{N-m})}, \quad t\in I, \end{aligned}$$

satisfies part (A) of Theorem 1.1 along *any* infinitesimal sequence $(t_k)_{k\in \mathbb {N}}\subset I$, with $\nu _t^{(m,2)}{:}{=}\rho _t^{(m,2)}{\mathscr {L}}^N\overset{\star }{\rightharpoonup }\delta _0$ in ${\mathscr {M}}_\textrm{loc}(\mathbb {R}^N)$ as $t\rightarrow 0^+$ and limit Dirichlet energy

$$\begin{aligned} {\mathscr {D}}^{(N-m,2)}_p(u) = c_{N-m,2}\int _{{\mathbb {S}}^{N-m,2}}\Vert \sigma \cdot Du\Vert _{L^p}^p\,\textrm{d}{\mathscr {H}}^{N-m-1}(\sigma ), \quad u\in \mathcal {S}^{p}(\mathbb {R}^N), \end{aligned}$$

for any $p\in [1,\infty )$, where now

$$\begin{aligned} {\mathbb {S}}^{N-m-1,2}=\left\{ x\in \mathbb {R}^N: |x''|=1\right\} , \quad c_{N-m,2}={\mathscr {H}}^{N-m-1}({\mathbb {S}}^{N-m-1,2})^{-1}. \end{aligned}$$

Now consider the family $(\rho _t)_{t\in I}$ defined as

$$\begin{aligned} \rho _t = {\left\{ \begin{array}{ll} \rho _t^{(1,1)} &  \text {for }t=\frac{1}{k} \text { with }k \text { odd }, \\ \rho _t^{(N-1,2)} &  \text {for }t=\frac{1}{k} \text { with }k \text { even}, \\ 0 &  \text {otherwise}. \end{array}\right. } \end{aligned}$$

From the observations made above we have that $(\rho _t)_{t\in I}$ satisfies part (A) in Theorem 1.1 along the sequence $t_k=\frac{1}{k}$, $k\in \mathbb {N}$, and moreover, given that obviously we have that $c_{1,1}=c_{N-1,2}$,

$$\begin{aligned} \lim _{\begin{array}{c} k\rightarrow \infty \\ k\,\text {odd} \end{array}} {\mathscr {F}}_{t_k,p}(u) = \Vert \mathrm e_1\cdot Du\Vert _{L^p}^p \quad \text {and} \quad \lim _{\begin{array}{c} k\rightarrow \infty \\ k\,\text {even} \end{array}} {\mathscr {F}}_{t_k,p}(u) = \Vert \mathrm e_N\cdot Du\Vert _{L^p}^p \end{aligned}$$

for every $u\in \mathcal {S}^{p}(\mathbb {R}^N)$ and $p\in [1,\infty )$. This means that, for part (B) of Theorem 1.1 to hold, one must pass to a subsequence of $(t_k)_{k\in \mathbb {N}}$ in general.

## Proof of Lemma 1.3

In this section we specialize our analysis to a particular class of families of kernels.

### Special Kernels

We let $p\in [1,\infty )$ and $K:\mathbb {R}^N\rightarrow [0,\infty ]$ be a measurable function such that $K\not \equiv 0$ and $|\cdot |^p\,K\in L^1_\textrm{loc}(\mathbb {R}^N)$. We set

4.1 $$\begin{aligned} m_{K,p}(R) = \int _{B_R}|x|^p\,K(x)\,\textrm{d}x\in [0,\infty ) \end{aligned}$$

for all $R>0$. We note that, since $K\not \equiv 0$, there exists $R_0>0$ such that $m_{K,p}(R)>0$ for all $R\ge R_0$. We let $\beta :I\rightarrow (0,\infty )$ be a Borel function such that

4.2 $$\begin{aligned} \lim _{t\rightarrow 0^+}\beta (t)=\infty , \end{aligned}$$

and we set

4.3 $$\begin{aligned} \phi _{K,\beta ,p}(t) = \frac{m_{K,p}(\beta (t))}{\beta (t)^p} \end{aligned}$$

for all $t>0$. Owing to (4.2) and the previous definition of $R_0>0$, we can find $t_0\in (0,1)$ such that $\phi _{K,\beta ,p}(t)>0$ for all $t\in (0,t_0)$. Finally, we let $(K_t)_{t\in I}$ be given by

$$\begin{aligned} K_t(x) = \beta (t)^N K(\beta (t)x) \end{aligned}$$

for each $t>0$ and $x\in \mathbb {R}^N$. Recalling the previous definition of $t_0\in (0,1)$, we can hence consider the following special family of kernels $(\rho _t)_{t\in I_0}\subset L^1_\textrm{loc}(\mathbb {R}^N)$, $I_0=(0,t_0)$, depending on *p*, *K* and $\beta$, given by

4.4 $$\begin{aligned} \rho _t(x) = \frac{|x|^p K_t(x)}{\phi _{K,\beta ,p}(t)} \end{aligned}$$

for $t\in I_0$ and $x\in \mathbb {R}^N$. We observe that, for the family (4.4), the functional ${\mathscr {F}}_{t,p}$ in (2.8) can be rewritten as

4.5 $$\begin{aligned} {\mathscr {F}}_{t,p}^{K,\beta }(u) = \frac{1}{\phi _{K,\beta ,p}(t)} \int _{\mathbb {R}^N}\int _{\mathbb {R}^N} |u(x)-u(y)|^p \, K_t(x-y)\,\textrm{d}x\,\textrm{d}y \end{aligned}$$

for each $t\in I_0$. Without loss of generality, for simplicity we will assume that $t_0=1$ and thus $I_0=I$ as usual and, unless required for better clarity, we will omit the dependence on *K* and $\beta$ in the quantities of interest to keep the notation short.

### Convergence to Local Energies

We now apply Theorem 1.1 to the special family of kernels given by (4.4). To this aim, we state the following result, which rephrases Lemma 2.9 for the special family in (4.4). A similar result has been discussed in [61, Ex. 2].

#### Proposition 4.1

Let $p\in [1,\infty )$, $K\not \equiv 0$ and $\beta :I\rightarrow (0,\infty )$ be as above. The measures $(\mu _t^\delta )_{t,\delta \in I}$ in (2.4) in Lemma 2.9 corresponding to the family $(\rho _t)_{t\in I}$ in (4.4) are given by

$$\begin{aligned} \mu _t^\delta (E) = \int _{E}\left( \int _0^{\beta (t)\delta } r^{N+p-1}K(\sigma r)\,\textrm{d}r\right) \frac{\,\textrm{d}{\mathscr {H}}^{N-1}(\sigma )}{m_{K,p}(\beta (t))} \end{aligned}$$

for every ${\mathscr {H}}^{N-1}$-measurable set $E\subset {\mathbb {S}}^{N-1}$. Moreover, if

4.6 $$\begin{aligned} |\cdot |^p\,K\in L^1(\mathbb {R}^N), \end{aligned}$$

then the following hold:

i $\mu _t^\delta \overset{\star }{\rightharpoonup }\theta _{K,p}\,{\mathscr {H}}^{N-1}$ in ${\mathscr {M}}({\mathbb {S}}^{N-1})$ as $t\rightarrow 0^+$ for $\delta >0$, where $\theta _{K,p}:{\mathbb {S}}^{N-1}\rightarrow [0,\infty ]$ is given by
  4.7 $$\begin{aligned} \theta _{K,p} (\sigma ) = \frac{\displaystyle \int _0^\infty r^{N+p-1}\,K(\sigma r)\,\textrm{d}r}{\Vert |\cdot |^p\,K\Vert _{L^1}} \quad \text {for } {\mathscr {H}}^{N-1}-\text {a.e. } \ \sigma \in {\mathbb {S}}^{N-1}; \end{aligned}$$
ii the measures $(\nu _t)_{t\in I}$, defined as $\nu _t=\rho _t\,{\mathscr {L}}^N$ for every $t\in I$, where the family $(\rho _t)_{t\in I}$ is as in (4.4), satisfy $\nu _t\overset{\star }{\rightharpoonup }\delta _0$ in ${\mathscr {M}}_\textrm{loc}(\mathbb {R}^N)$ as $t\rightarrow 0^+$;
iii if *K* is radially symmetric, then the family $(\rho _t)_{t\in I}$ in (4.4) has maximal rank and $\theta _{K,p}(\sigma )=\frac{1}{N\omega _N}$ for every ${\mathscr {H}}^{N-1}$-a.e. $\sigma \in {\mathbb {S}}^{N-1}$, so that
  4.8 $$\begin{aligned} \int _{{\mathbb {S}}^{N-1}} \Vert \sigma \cdot Du\Vert ^p_{L^p}\,\theta _{K,p}(\sigma )\,\textrm{d}{\mathscr {H}}^{N-1}(\sigma ) = \frac{2}{N} \, \frac{\Gamma \left( \frac{N+1}{2}\right) \Gamma \left( \frac{p+1}{2}\right) }{\Gamma \left( \frac{N+p}{2}\right) } \, \Vert Du\Vert _{L^p}^p\nonumber \\ \end{aligned}$$
  for every $$u\in {\mathcal {S}^{p}}(\mathbb {R}^N)$$.

#### Proof

Let us first observe that, by (4.2) and (4.6), we have

4.9 $$\begin{aligned} \lim _{t\rightarrow 0^+} m_{K,p}(\beta (t)) = \Vert |\cdot |^p\,K\Vert _{L^1}\in (0,\infty ). \end{aligned}$$

We can now briefly prove each statement separately.

*Proof of* (i). Given $\delta >0$ and $\varphi \in C({\mathbb {S}}^{N-1})$, we can compute

$$\begin{aligned} \begin{aligned} \lim _{t\rightarrow 0^+} \int _{{\mathbb {S}}^{N-1}}\varphi (\sigma )\,\textrm{d}\mu _t^\delta (\sigma )&= \lim _{t\rightarrow 0^+} \frac{1}{m_{K,p}(\beta (t))} \int _{{\mathbb {S}}^{N-1}}\varphi (\sigma ) \int _0^{\beta (t)\delta }r^{N+p-1}K(\sigma r)\,\textrm{d}r\,\textrm{d}{\mathscr {H}}^{N-1}(\sigma ) \\&= \frac{1}{\Vert |\cdot |^p\,K\Vert _{L^1}} \int _{{\mathbb {S}}^{N-1}}\varphi (\sigma ) \int _0^{\infty }r^{N+p-1}K(\sigma r) \,\textrm{d}r \,\textrm{d}{\mathscr {H}}^{N-1}(\sigma ) \\&= \int _{{\mathbb {S}}^{N-1}}\varphi (\sigma ) \, \theta _{K,p}(\sigma ) \,\textrm{d}{\mathscr {H}}^{N-1}(\sigma ) \end{aligned} \end{aligned}$$

by (4.2), (4.9), the Dominated Convergence Theorem, and Fubini’s Theorem.

*Proof of* (ii). In view of (4.9), we infer that

$$\begin{aligned} \lim _{t\rightarrow 0^+} \nu _t(\mathbb {R}^N) = \lim _{t\rightarrow 0^+} \Vert \rho _t\Vert _{L^1} = \lim _{t\rightarrow 0^+} \frac{\Vert |\cdot |^p\,K\Vert _{L^1}}{m_{K,p}(\beta (t))} = 1. \end{aligned}$$

Moreover, if $\varphi \in C_c(\mathbb {R}^N)$, then, by changing variables, we have

4.10 $$\begin{aligned} \begin{aligned} \lim _{t\rightarrow 0^+} \int _{\mathbb {R}^N}\varphi \,\,\textrm{d}\nu _t = \lim _{t\rightarrow 0^+} \frac{1}{m_{K,p}(\beta (t))} \int _{\mathbb {R}^N}\varphi \left( \frac{x}{\beta (t)}\right) |x|^p\,K(x)\,\textrm{d}x = \varphi (0) \end{aligned}\nonumber \\ \end{aligned}$$

by (4.2), (4.9) and the Dominated Convergence Theorem, owing to (4.6).

*Proof of* (iii). Formula (4.8) is well known, see [42, 61] for instance. Thus, we just focus on the maximal rank property. We shall prove that Definition 2.8 is satisfied for the canonical basis of $\mathbb {R}^N$, $\mathrm e_1,\dots ,\mathrm e_N\in {\mathbb {S}}^{N-1}$, and any $\tau \in I$ sufficiently small. Indeed, by radial symmetry, we have

$$\begin{aligned} \int _{B_\delta \,\cap \,{\mathcal {C}}_\tau (\mathrm e_i)}\rho _t(z)\,\textrm{d}z = \int _{B_\delta \,\cap \,{\mathcal {C}}_\tau (\mathrm e_1)}\rho _t(z)\,\textrm{d}z \end{aligned}$$

for all $i\in \left\{ 1,\dots ,N\right\}$. Arguing as in (4.10), we can compute

$$\begin{aligned} \int _{B_\delta \,\cap \,{\mathcal {C}}_\tau (\mathrm e_1)}\rho _t(z)\,\textrm{d}z = \frac{1}{m_{K,p}(\beta (t))} \int _{B_{\delta \beta (t)}\,\cap \,{\mathcal {C}}_\tau (\mathrm e_1)} |x|^p\,K(x)\,\textrm{d}x. \end{aligned}$$

Therefore, by combining the above equalities, we get that

$$\begin{aligned} \liminf _{t\rightarrow 0^+} \int _{B_\delta \,\cap \,{\mathcal {C}}_\tau (\mathrm e_i)}\rho _t(z)\,\textrm{d}z = \lim _{t\rightarrow 0^+} \frac{1}{m_{K,p}(\beta (t))} \int _{B_{\delta \beta (t)}\,\cap \,{\mathcal {C}}_\tau (\mathrm e_1)} |x|^p\,K(x)\,\textrm{d}x = c_{K,p} \end{aligned}$$

by (4.2), (4.9) and the Monotone Convergence Theorem, where

$$\begin{aligned} c_{K,p} = \frac{1}{\Vert |\cdot |^p\,K\Vert _{L^1}} \int _{{\mathcal {C}}_\tau (\mathrm e_1)} |x|^p\,K(x)\,\textrm{d}x\in (0,1) \end{aligned}$$

and the validity of Definition 2.8 readily follows, concluding the proof. $\square$

We are now ready to apply Theorem 1.1 to the special family in (4.4). We remark that Lemma 4.2 below was already implicitly given in [61], although the main results of [61] are stated on bounded open subsets of $\mathbb {R}^N$ with Lipschitz boundary.

#### Theorem 4.2

Let $p\in [1,\infty )$, $K\not \equiv 0$ and $\beta :I\rightarrow (0,\infty )$ be as above. If (4.6) holds, then the limit

$$\begin{aligned}&\lim _{t\rightarrow 0^+} \beta (t)^p \int _{\mathbb {R}^N}\int _{\mathbb {R}^N} |u(x)-u(y)|^p \, K_t(x-y)\,\textrm{d}x\,\textrm{d}y \\&= \int _{{\mathbb {S}}^{N-1}} \Vert \sigma \cdot Du\Vert ^p_{L^p}\,\theta _{K,p}(\sigma )\,\textrm{d}{\mathscr {H}}^{N-1}(\sigma ), \end{aligned}$$

where $\theta _{K,p}:{\mathbb {S}}^{N-1}\rightarrow [0,\infty ]$ is as in (4.7), holds for $u\in {\mathcal {S}^{p}}(\mathbb {R}^N)$ pointwise and in the $\Gamma$-sense with respect to the $L^p$ topology, and the functionals on the right-hand side are coercive. If, in addition, *K* is radially symmetric, then the limit

$$\begin{aligned}&\lim _{t\rightarrow 0^+} \beta (t)^p \int _{\mathbb {R}^N}\int _{\mathbb {R}^N} |u(x)-u(y)|^p \, K_t(x-y)\,\textrm{d}x\,\textrm{d}y \\&= \frac{2}{N} \, \frac{\Gamma \left( \frac{N+1}{2}\right) \Gamma \left( \frac{p+1}{2}\right) }{\Gamma \left( \frac{N+p}{2}\right) \,\Vert |\cdot |^p K\Vert _{L^1}} \, \Vert Du\Vert _{L^p}^p \end{aligned}$$

holds for $u\in {\mathcal {S}^{p}}(\mathbb {R}^N)$ pointwise and in the $\Gamma$-sense with respect to the $L^p$ topology, and the functionals on the right-hand side are coercive.

#### Proof

The statement directly follows by combining Theorem 3.1 with the properties collected in Lemma 4.1. We omit the simple computations. $\square$

### Compactness

We now complete the asymptotic analysis of the energies (4.5) relative to the special family (4.4) achieved in Lemma 4.2 by proving Lemma 1.3. To this aim, we need to introduce the following terminology.

#### Definition 4.3

(*Local precompactness*) Let $p\in [1,\infty )$. A set ${\mathcal {X}}\subset L^p(\mathbb {R}^N)$ is locally precompact in $L^p(\mathbb {R}^N)$ if ${\mathcal {X}}$ is precompact in $L^p(E)$ for every compact set $E\subset \mathbb {R}^N$.

For the proof of Lemma 1.3, we need the following preliminary result, which generalizes (the proof of) [15, Th. 3.5] to every $p\in [1,\infty )$.

#### Proposition 4.4

Let $p\in [1,\infty )$, $K\not \equiv 0$ and $\beta :I\rightarrow (0,\infty )$ be as above and assume (4.6). If $u\in L^p(\mathbb {R}^N)$ and $t>0$, then there exists $v_t\in {\mathcal {S}^{p}}(\mathbb {R}^N)$ such that

$$\begin{aligned} \Vert v_t-u\Vert _{L^p} \le C_{K,p} \, {\mathscr {F}}^K_{t,p}(u) \, \beta (t)^{-p} \quad \text {and} \quad \Vert \nabla v_t\Vert _{L^p} \le C_{K,p} \, {\mathscr {F}}^K_{t,p}(u), \end{aligned}$$

where $C_{K,p}>0$ depends on *K* and *p* only.

In the proof of Proposition 4.4 we need the following estimate, which revisits the one in [15, Lem. 3.4] for every $p\in [1,\infty )$. (we omit its proof, since it follows by a simple application of Tonelli’s Theorem):

#### Lemma 4.5

Let $p\in [1,\infty )$. If $G\in L^1(\mathbb {R}^N)$ is a non-negative function, then

$$\begin{aligned} \int _{\mathbb {R}^N}\Vert u(\cdot +z)-u\Vert _{L^p}^p \, (G* G)(z) \,\textrm{d}z \le 2^p \, \Vert G\Vert _{L^1} \int _{\mathbb {R}^N}\Vert u(\cdot +z)-u\Vert _{L^p}^p \, G(z) \,\textrm{d}z \end{aligned}$$

for every $$u\in L^p(\mathbb {R}^N)$$.

#### Proof of Proposition 4.4

Since $K\not \equiv 0$ and $|\cdot |^p\, K\in L^1(\mathbb {R}^N)$ by (4.6), we have that the function $G=\min \left\{ K,1\right\}$ satisfies $G\in L^1\cap L^\infty (\mathbb {R}^N){\setminus }\left\{ 0\right\}$, with

$$\begin{aligned} \Vert G\Vert _{L^1} \le |B_1| + \int _{B_1^c}|x|^p\,K(x)\,\textrm{d}x \le |B_1| + \Vert |\cdot |^p K\Vert _{L^1}. \end{aligned}$$

Hence the function $G*G$ is non-negative, continuous, and strictly positive on a non-empty open set in $\mathbb {R}^N$. Thus, we can find a non-negative function $\varphi \in \operatorname {Lip}_c(\mathbb {R}^N){\setminus }\left\{ 0\right\}$ such that

4.11 $$\begin{aligned} \varphi \le G* G \quad \text {and} \quad |\nabla \varphi |\le G* G. \end{aligned}$$

We hence set

$$\begin{aligned} G_t(x)=\beta (t)^NG(\beta (t)x), \quad \varphi _t=\frac{\beta (t)^N\varphi (\beta (t)x)}{\Vert \varphi \Vert _{L^1}} \end{aligned}$$

for every $t>0$ and $x\in \mathbb {R}^N$. We note that $\Vert G_t\Vert _{L^1}=\Vert G\Vert _{L^1}$, $\Vert \varphi _t\Vert _{L^1}=1$ and, moreover, owing to (4.11),

4.12 $$\begin{aligned} \varphi _t \le \frac{G_t* G_t}{\Vert \varphi \Vert _{L^1}} \quad \text {and} \quad |\nabla \varphi _t| \le \frac{G_t* G_t}{\Vert \varphi \Vert _{L^1}} \, \beta (t) \end{aligned}$$

for every $t>0$. Finally, given $u\in L^p(\mathbb {R}^N)$, we set $v_t=u*\varphi _t$ for every $t>0$ and we note that $v_t\in {\mathcal {S}^{p}}(\mathbb {R}^N)$ for every $t>0$. Owing to Jensen’s inequality, (4.12), Lemma 4.5 and the definitions in (4.1) and (4.3), we can estimate

$$\begin{aligned} \begin{aligned} \begin{aligned} \Vert v_t&-u\Vert _{L^p}^p \le \int _{\mathbb {R}^N}\Vert u(\cdot +z)-u\Vert _{L^p}^p\,\varphi _t(z)\,\text {d}z \le \frac{1}{\Vert \varphi \Vert _{L^1}} \int _{\mathbb {R}^N}\Vert u(\cdot +z)-u\Vert _{L^p}^p\,(G_t* G_t)(z)\,\text {d}z\\  &\le \frac{2^p\Vert G_t\Vert _{L^1}}{\Vert \varphi \Vert _{L^1}} \int _{\mathbb {R}^N}\Vert u(\cdot +z)-u\Vert _{L^p}^p\,G_t(z)\,\text {d}z \le \frac{2^p\Vert G\Vert _{L^1}}{\Vert \varphi \Vert _{L^1}} \, \int _{\mathbb {R}^N}\Vert u(\cdot +z)-u\Vert _{L^p}^p\,K_t(z)\,\text {d}z \\  &= \frac{2^p\Vert G\Vert _{L^1}}{\Vert \varphi \Vert _{L^1}} \, {\mathscr {F}}^K_{p,t}(u) \, \phi _{K,p}(t) \le \frac{2^p\Vert G\Vert _{L^1}}{\Vert \varphi \Vert _{L^1}} \, \Vert |\cdot |^p\,K\Vert _{L^1} \, {\mathscr {F}}^K_{p,t}(u) \, \beta (t)^{-p}. \end{aligned} \end{aligned} \end{aligned}$$

Moreover, since, for all $x\in \mathbb {R}^N$, by the Divergence Theorem, we can write,

$$\begin{aligned} \nabla v_t(x) = \int _{\mathbb {R}^N}u(x-z)\,\nabla \varphi _t(z)\,\textrm{d}z = \int _{\mathbb {R}^N}(u(x-z)-u(x))\,\nabla \varphi _t(z)\,\textrm{d}z, \end{aligned}$$

we similarly get that

$$\begin{aligned} \begin{aligned} \begin{aligned} \Vert \nabla v_t\Vert _{L^p}^p&\le \Vert \nabla \varphi _t\Vert _{L^1}^{p-1} \int _{\mathbb {R}^N}\Vert u(\cdot +z)-u\Vert _{L^p}^p\,|\nabla \varphi _t(z)|\,\text {d}z \\  &\le \Vert \nabla \varphi \Vert _{L^1}^{p-1} \, \frac{\beta (t)^p}{\Vert \varphi \Vert _{L^1}^p} \int _{\mathbb {R}^N}\Vert u(\cdot +z)-u\Vert _{L^p}^p\,(G_t* G_t)(z)\,\text {d}z \\  &\le 2^p\Vert G\Vert _{L^1} \, \frac{\Vert \nabla \varphi \Vert _{L^1}^{p-1}}{\Vert \varphi \Vert _{L^1}^p} \, \Vert |\cdot |^p K\Vert _{L^1} \, {\mathscr {F}}^K_{p,t}(u)\end{aligned} \end{aligned} \end{aligned}$$

for every $t>0$, yielding the conclusion. $\square$

#### Proof of Lemma 1.3

For convenience, we let

$$\begin{aligned} M=\sup _{k\in \mathbb {N}} \big ( \Vert u_k\Vert _{L^p} + {\mathscr {F}}_{t_k,p}(u_k) \big )<\infty . \end{aligned}$$

By Proposition 4.4 we can find $v_k=v_{t_k}\in {\mathcal {S}^{p}}(\mathbb {R}^N)$ such that $\Vert v_k-u_k\Vert _{L^p} \le C\beta (t_k)^{-p}$ and $\Vert \nabla v_k\Vert _{L^p} \le C$ for every $k\in \mathbb {N}$, where $C>0$ depends on *K*, *p* and *M* only. In particular, the sequence $(v_k)_{k\in \mathbb {N}}$ is bounded in ${\mathcal {S}^{p}}(\mathbb {R}^N)$ and thus we can find a subsequence $(v_{k_j})_{j\in \mathbb {N}}$ and $u\in {\mathcal {S}^{p}}(\mathbb {R}^N)$ such that $v_{k_j}\rightarrow u$ in $L^p_\textrm{loc}(\mathbb {R}^N)$ as $j\rightarrow \infty$. Since $\beta (t_k)\rightarrow \infty$ as $k\rightarrow \infty$, we also get that $u_k\rightarrow u$ in $L^p_\textrm{loc}(\mathbb {R}^N)$ as $k\rightarrow \infty$. A similar argument proves that any $L^p_\textrm{loc}(\mathbb {R}^N)$ limit of $(u_k)_{k\in \mathbb {N}}$ belongs to ${\mathcal {S}^{p}}(\mathbb {R}^N)$. $\square$

For future convenience, we complete Lemma 1.3 by recalling the following result, which is a consequence of [62, Ths. 1.2 and 1.3] (we also refer to the discussion in [62, Sec. 2]).

#### Proposition 4.6

Let $p\in [1,\infty )$. If $(\lambda _k)_{k\in \mathbb {N}}\subset I$ is infinitesimal and $(u_k)_{k\in \mathbb {N}}\subset L^p(\mathbb {R}^N)$ is such that

$$\begin{aligned} \sup _{k\in \mathbb {N}} \left( \Vert u_k\Vert _{L^p} + \int _{\mathbb {R}^N}\frac{\Vert u_k(\cdot +z)-u_k\Vert _{L^p}^p}{|\log \lambda _k|\,(\lambda _k+|z|)^{N+p}}\,\textrm{d}z \right) <\infty , \end{aligned}$$

then $(u_k)_{k\in \mathbb {N}}$ is locally precompact in $L^p(\mathbb {R}^N)$ and any of its $L^p_\textrm{loc}(\mathbb {R}^N)$ limits is in ${\mathcal {S}^{p}}(\mathbb {R}^N)$.

#### Proof

The results follows from [62, Ths. 1.2 and 1.3]. Indeed, it is enough to consider the non-negative radial kernels $(\rho _k)_{k\in \mathbb {N}}$ defined as

$$\begin{aligned} \rho _k(z) = \frac{c_k|z|^p\,\chi _{B_1}(z)}{|\log \lambda _k|\,(\lambda _k+|z|)^{N+p}}, \end{aligned}$$

for all $z\in \mathbb {R}^N$ and $k\in \mathbb {N}$, where

$$\begin{aligned} c_k = \int _{B_1} \frac{|z|^p}{|\log \lambda _k|\,(\lambda _k+|z|)^{N+p}}\,\textrm{d}z \end{aligned}$$

is a renormalization constant such that $\displaystyle \int _{\mathbb {R}^N}\rho _k(z)\,\textrm{d}z=1$ for all $k\in \mathbb {N}$. Since

$$\begin{aligned} 0< \inf _{k\in \mathbb {N}}c_k \le \sup _{k\in \mathbb {N}}c_k < \infty , \end{aligned}$$

the kernels $(\rho _k)_{k\in \mathbb {N}}$ satisfy the properties in [62, Eq. (2)], and also the additional property in [62, Eq. (8)] for $N=1$. We omit the plain details. $\square$

## Proof of Theorems 1.4 and 1.5

We now pass to the proof of the non-local stability results, Theorems 1.4 and 1.5.

### Proof of Theorem 1.4

If $u\in W^{\kappa ,p}(\mathbb {R}^N)$, then $z\mapsto \Vert u(\cdot +z)-u\Vert _{L^p}^p\,\kappa (z)\in L^1(\mathbb {R}^N)$ and thus, by (1.8), we can apply the Dominated Convergence Theorem to get that

$$\begin{aligned} \lim _{t\rightarrow 0^+} {\mathscr {F}}_{t,p}(u) = \int _{\mathbb {R}^N}\Vert u(\cdot +z)-u\Vert _{L^p}^p\,\kappa (z)\,\textrm{d}z = [u]_{W^{\kappa ,p}}. \end{aligned}$$

Moreover, if $(t_k)_{k\in \mathbb {N}}\subset I$ is infinitesimal and $(u_k)_{k\in \mathbb {N}}\subset L^p(\mathbb {R}^N)$ is such that $u_k\rightarrow u$ in $L^p(\mathbb {R}^N)$ as $k\rightarrow \infty$, then by (1.9) we can apply Fatou’s Lemma to get

$$\begin{aligned} \begin{aligned} \liminf _{k\rightarrow \infty }{\mathscr {F}}_{t_k,p}(u_k)&\ge \int _{\mathbb {R}^N}\lim _{k\rightarrow \infty } \Vert u_k(\cdot +z)-u_k\Vert _{L^p}^p\,\frac{\rho _{t_k}(z)}{|z|^p} \,\textrm{d}z \\&= \int _{\mathbb {R}^N} \Vert u(\cdot +z)-u\Vert _{L^p}^p\,\kappa (z) \,\textrm{d}z = [u]_{W^{\kappa ,p}}^p. \end{aligned} \end{aligned}$$

In particular, if $\liminf \limits _{k\rightarrow \infty }{\mathscr {F}}_{t_k,p}(u_k)<\infty$, then $u\in W^{\kappa ,p}(\mathbb {R}^N)$. The proof is complete.

$$\square $$

For the proof of Theorem 1.5 we need the following simple estimate exploiting (1.10).

### Lemma 5.1

Let $p\in [1,\infty )$ and $(\rho _t)_{t\in I}\subset L^1_\textrm{loc}(\mathbb {R}^N)$. If (1.10) holds, then for every $\varepsilon >0$ there exists $\delta >0$ such that, letting $\eta _\delta =\chi _{B_\delta }/|B_\delta |$,

$$\begin{aligned} \Vert \eta _\delta *u-u\Vert _{L^p}^p \le \frac{\varepsilon }{|B_1|}\,{\mathscr {F}}_{t,p}(u) \end{aligned}$$

for every $t\in (0,\delta )$ and $u\in L^p(\mathbb {R}^N)$.

### Proof

Let $u\in L^p(\mathbb {R}^N)$ and $\varepsilon ,\delta >0$ be as in (1.10). By Jensen’s inequality, we have

$$\begin{aligned} \begin{aligned} \Vert \eta _\delta *u-u\Vert _{L^p}^p \le \frac{1}{|B_\delta |}\! \int _{B_\delta }\!\! \Vert u(\cdot -z)-u\Vert _{L^p}^p\,\textrm{d}z = \frac{1}{|B_1|}\! \int _{B_\delta } \frac{\Vert u(\cdot +z)-u\Vert _{L^p}^p}{\delta ^N}\,\textrm{d}z. \end{aligned} \end{aligned}$$

In virtue of (1.10), we thus infer that

$$\begin{aligned} \begin{aligned} \Vert \eta _\delta *u-u\Vert _{L^p}^p \le \frac{\varepsilon }{|B_1|} \int _{B_\delta } \Vert u(\cdot +z)-u\Vert _{L^p}^p \, \frac{\rho _t(z)}{|z|^p}\,\textrm{d}z = \frac{\varepsilon }{|B_1|} \,{\mathscr {F}}_{t,p}(u), \end{aligned} \end{aligned}$$

concluding the proof. $\square$

### Proof of Theorem 1.5

By assumption, the sequence $(u_k)_{k\in \mathbb {N}}$ is bounded in $L^p(\mathbb {R}^N)$. Thus, letting $\eta _\delta =\chi _{B_\delta }/{|B_\delta |}\in L^1(\mathbb {R}^N)$ for $\delta >0$, by [24, Cor. 4.28] the sequence $(\eta _\delta *u_k)_{k\in \mathbb {N}}$ is locally precompact in $L^p(\mathbb {R}^N)$ for each $\delta >0$. As a consequence, the sequence $(\eta _\delta *u_k)_{k\in \mathbb {N}}$ is totally bounded in $L^p(E)$ for every compact set $E\subset \mathbb {R}^N$. By Lemma 5.1, also the sequence $(u_k)_{k\in \mathbb {N}}$ is totally bounded in $L^p(E)$ for every compact set $E\subset \mathbb {R}^N$, which implies that the sequence $(u_k)_{k\in \mathbb {N}}$ is locally precompact in $L^p(\mathbb {R}^N)$. Finally, if *u* is an $L^p_\textrm{loc}(\mathbb {R}^N)$ limit of $(u_k)_{k\in \mathbb {N}}$, then, up to subsequences, by Fatou’s Lemma we have

$$\begin{aligned} \begin{aligned} \sup _{k\in \mathbb {N}}{\mathscr {F}}_{t_k,p}(u_k)&\ge \!\! \liminf _{k\rightarrow \infty }{\mathscr {F}}_{t_k,p}(u_k) \ge \int _{\mathbb {R}^N}\liminf _{k\rightarrow \infty } \left( \Vert u_k(\cdot +z)-u_k\Vert _{L^p}^p\,\frac{\rho _{t_k}(z)}{|z|^p} \right) \! \,\textrm{d}z \\&\ge \int _{\mathbb {R}^N} \Vert u(\cdot +z)-u\Vert _{L^p}^p\,\kappa (z) \,\textrm{d}z = [u]_{W^{\kappa ,p}}^p, \end{aligned} \end{aligned}$$

showing that $u\in W^{\kappa ,p}(\mathbb {R}^N)$ and concluding the proof. $\square$

## Application to Heat Kernels

In this section we apply the results of Sects. 4 and 5 to families $(\rho _t)_{t\in I}$ induced by heat-type kernels, proving Theorems 1.6 and 1.7.

### Heat Kernel

We let $(\textsf{h}_t)_{t>0}:\mathbb {R}^N\rightarrow (0,\infty )$ be the *heat kernel*, which is given by

6.1 $$\begin{aligned} \textsf{h}_t(x)=\frac{e^{-\frac{|x|^2}{4t}}}{(4\pi t)^{\frac{N}{2}}}, \end{aligned}$$

for all $x\in \mathbb {R}^N$ and $t>0$. Given $p\in [1,\infty ]$, we define the *heat semigroup*

$$\begin{aligned} (\textsf{H}_t)_{t>0}:L^p(\mathbb {R}^N)\rightarrow L^p(\mathbb {R}^N) \end{aligned}$$

by letting

6.2 $$\begin{aligned} \textsf{H}_tu=\textsf{h}_t*u \end{aligned}$$

for all $u\in L^p(\mathbb {R}^N)$ and $t>0$. Note that (6.2) makes sense by Young’s inequality, since $\textsf{h}_t\in L^1(\mathbb {R}^N)$ for all $t>0$ due to (6.1).

We can now deal with the proof of Theorem 1.6.

#### Proof of Theorem 1.6

Letting $K(x)=\textsf{h}_1(x)$ for all $x\in \mathbb {R}^N$ and $\beta (t)=t^{-\frac{1}{2}}$ for all $t>0$, we get that $K_t(x)=\textsf{h}_t(x)$ for all $x\in \mathbb {R}^N$ and $t>0$. By radial symmetry, we can compute that

$$\begin{aligned} \Vert |\cdot |^p\,K\Vert _{L^1} = \frac{2^{p-1}N}{\sqrt{\pi }}\,\frac{\Gamma \left( \frac{N+p}{2}\right) }{\Gamma \left( \frac{N+1}{2}\right) }. \end{aligned}$$

Since $|\cdot |^p\,K\in L^1(\mathbb {R}^N)$ for every $p\in [1,\infty )$ due to (6.1), the conclusion hence follows from Theorems 4.2 and 1.3. We omit the simple computations. $\square$

### Fractional Heat Kernel

Given $s\in (0,1)$, we let $(\textsf{h}_t^s)_{t>0}:\mathbb {R}^n\rightarrow (0,\infty )$ be the *fractional heat kernel*. For $s=\frac{1}{2}$, it is known that

6.3 $$\begin{aligned} \textsf{h}_t^{\frac{1}{2}}(x) = \frac{\Gamma \left( \frac{N+1}{2}\right) }{\pi ^{\frac{N+1}{2}}} \, \frac{t}{\left( t^2+|x|^2\right) ^{\frac{N+1}{2}}} \end{aligned}$$

for all $x\in \mathbb {R}^N$ and $t>0$, see [68, Eq. (2.2)]. For $s\in (0,1)$, $s\ne \frac{1}{2}$, the heat kernel does not have an explicit formula. Anyway, it is a smooth, positive, radially symmetric probability function obeying the scaling law

6.4 $$\begin{aligned} \begin{aligned} \textsf{h}_t^s(x)=t^{-\frac{N}{2s}}\,\textsf{h}_1^s\left( t^{-\frac{1}{2s}}x\right) \end{aligned} \end{aligned}$$

for all $x\in \mathbb {R}^N$ and $t>0$. Moreover, by [18, Th. 2.1], we have that

$$\begin{aligned} \lim _{x\rightarrow \infty } |x|^{N+2s}\,\textsf{h}_1^s(x) = \zeta _{N,s}, \end{aligned}$$

with

6.5 $$\begin{aligned} \zeta _{N,s} = \frac{s\,4^s}{\pi ^{\frac{N}{2}}} \, \frac{\Gamma \left( \frac{N}{2}+s\right) }{\Gamma (1-s)}. \end{aligned}$$

Therefore, thanks to (6.4), we also get that

6.6 $$\begin{aligned} \lim _{t\rightarrow 0^+} \frac{\textsf{h}_t^s(x)}{t} = \frac{\zeta _{N,s}}{|x|^{N+2s}} \end{aligned}$$

for all $x\in \mathbb {R}^N\setminus \left\{ 0\right\}$, where $\zeta _{N,s}$ is as in (6.5), and that there exists $C_{N,s}>0$ such that

6.7 $$\begin{aligned} \frac{C_{N,s}^{-1}\,t}{\left( t^{\frac{1}{s}}+|x|^2\right) ^{\frac{N+2s}{2}}} \le \textsf{h}_t^s(x) \le \frac{C_{N,s}\,t}{\left( t^{\frac{1}{s}}+|x|^2\right) ^{\frac{N+2s}{2}}} \end{aligned}$$

for all $x\in \mathbb {R}^N$ and $t>0$. The fractional heat kernel enjoys the following relation with the integer heat kernel (6.1). Actually, such relation is a particular case of the general approach sketched in [19, (Sec. 1.1.4)]. For each $s\in (0,1)$, there exists a family of probability densities $(\eta _t^{s})_{t>0}$ on $(0,\infty )$ such that

6.8 $$\begin{aligned} \textsf{h}_t^s(x) = \int _0^\infty \textsf{h}_\tau (x)\,\eta _t^s(\tau )\,\textrm{d}\tau \quad \text {for all}\ x\in \mathbb {R}^N\ \text {and}\ t>0. \end{aligned}$$

As proved in [1, Sec. 2], the family $(\eta _t^{s})_{t>0}$ satisfies

6.9 $$\begin{aligned} \int _0^\infty \tau ^\alpha \,\eta _1^s(\tau )\,\textrm{d}\tau = \frac{\Gamma \left( 1-\frac{\alpha }{s}\right) }{\Gamma \left( 1-\alpha \right) } \end{aligned}$$

for all $$\alpha \in \left( -\infty ,s\right) $$.

As above, given $s\in (0,1)$ and $p\in [1,\infty ]$, we define the *fractional heat semigroup*

$$\begin{aligned} (\textsf{H}_t^s)_{t>0}:L^p(\mathbb {R}^N)\rightarrow L^p(\mathbb {R}^N) \end{aligned}$$

by letting

6.10 $$\begin{aligned} \textsf{H}_t^su=\textsf{h}_t^s*u \end{aligned}$$

for all $u\in L^p(\mathbb {R}^N)$ and $t>0$. We observe that (6.10) makes sense again by Young’s inequality, since $\textsf{h}_t^s\in L^1(\mathbb {R}^N)$ for all $t>0$ and $s\in (0,1)$, owing to the bounds in (6.7).

We can now deal with the proof of Theorem 1.7.

#### Proof of Theorem 1.7

Letting $K^s(x)=\textsf{h}_1^s(x)$ for all $x\in \mathbb {R}^N$ and $\beta _s(t)=t^{-\frac{s}{2}}$ for all $t>0$, we get that $K_t^s(x)=\textsf{h}_t^s(x)$ for all $x\in \mathbb {R}^N$ and $t>0$. We distinguish three cases.

*Case* $2s>p$. We have that $|\cdot |^p\,K^s\in L^1(\mathbb {R}^N)$, so the conclusion follows by Theorem 4.2. We just need to observe that, by radial symmetry, we can compute

$$\begin{aligned} \Vert |\cdot |^p\,K\Vert _{L^1} = \frac{2^{p-1}}{\sqrt{\pi }} \, \frac{\Gamma \left( \frac{N+p}{2}\right) }{\Gamma \left( \frac{N+1}{2}\right) } \, \frac{\Gamma \left( 1-\frac{p}{2s}\right) }{\Gamma \left( 1-\frac{p}{2}\right) } \end{aligned}$$

exactly as in [2, Lem. 4.1], thanks to the relation (6.8) and the formula (6.9). We omit the simple computations. For the compactness part, we can invoke Theorem 1.3.

*Case* $2s=p$. We have that $|\cdot |^{2\,s}\,K^s\notin L^1(\mathbb {R}^N)$. However, we can compute

$$\begin{aligned} m_s(R) = m_{K^s,2s}(R) = N\omega _N \int _0^R r^{N+2s-1}\,\textsf{h}_1^s(r\mathrm e_1)\,\textrm{d}r \quad \text {for all}\ R>0. \end{aligned}$$

Since

$$\begin{aligned} \frac{\textrm{d}}{\textrm{d}R} \int _0^R r^{N+2s-1}\,\textsf{h}_1^s(r\mathrm e_1)\,\textrm{d}r = R^{N+2s-1}\,\textsf{h}_1^s(R\mathrm e_1) \sim \frac{c_{N,s}}{R} \end{aligned}$$

as $R\rightarrow \infty$, where $c_{N,s}>0$ is as in (6.5), we must have that

$$\begin{aligned} m_s(R) \sim N\omega _N\,c_{N,s}\log R \end{aligned}$$

as $R\rightarrow \infty$. Hence, recalling the definition in (4.3), we deduce that

$$\begin{aligned} \phi _{K^s,2s,2s}(t) = t\,m_s\left( t^{-\frac{s}{2}}\right) \sim N\omega _N\,c_{N,s}\,\frac{s}{2}\,t|\log t| \end{aligned}$$

as $t\rightarrow 0^+$. The conclusion hence follows by observing that the family $(\rho _t)_{t>0}$ given by

$$\begin{aligned} \rho _t^s(x) = \frac{|x|^p\,\textsf{h}_t^s(x)}{t|\log t|}, \end{aligned}$$

for all $x\in \mathbb {R}^N$ and $t>0$, satisfies the properties in Theorem 1.1(A) (with no need of passing to a subsequence). For the compactness part, we can invoke Proposition 4.6.

*Case* $2s<p$. For the validity of the limit we can rely on Theorem 1.4, thanks to the limit in (6.6) and the bounds in (6.7), while the compactness part follows from Theorem 1.5. We omit the simple details. $\square$

#### Remark 6.1

(*On the constants in* Theorem 1.7*for* $p=1$) We observe that, for $p=1$, the constants in Theorem 1.7 were computed in [47] with a completely different method, based on the approach of [10]. We observe that our method is more direct and flexible, yielding the values of the constants for all $p\in [1,\infty )$.

### General Heat-Type Kernels

We conclude this section by generalizing [2, Th. 1.1], see Theorem 6.2 below. To state our result, we need to introduce some notation.

Let ${\mathscr {A}}$ be a set of indices and consider a family of non-negative continuous functions $(\textsf{h}_t^\alpha )_{t>0}:\mathbb {R}^N\rightarrow [0,\infty )$ for $\alpha \in {\mathscr {A}}$. Assume that $\textsf{h}_t^\alpha$ is radially symmetric with $\Vert \textsf{h}_t^\alpha \Vert _{L^1}=1$ for each $t>0$ and $\alpha \in {\mathscr {A}}$. Moreover, assume that there is $\beta >0$ such that

6.11 $$\begin{aligned} \textsf{h}_t^\alpha (x) = t^{-\beta N}\,\textsf{h}_1^\alpha (t^{-\beta }x) \end{aligned}$$

for all $x\in \mathbb {R}^N$, $t>0$ and $\alpha \in {\mathscr {A}}$. Given $p\in [1,\infty )$, we let

$$\begin{aligned} {\mathscr {A}}_{p} = \left\{ \alpha \in {\mathscr {A}}: |\cdot |^p\,\textsf{h}_1^\alpha \in L^1(\mathbb {R}^N)\right\} \end{aligned}$$

Moreover, given $\zeta >0$ and $C\ge 1$, we let ${\mathscr {B}}_p^{\zeta ,C}\subset {\mathscr {A}}$ be the subset of indices $\alpha \in {\mathscr {A}}$ such that

6.12 $$\begin{aligned} \lim _{|x|\rightarrow \infty }|x|^{N+p}\,\textsf{h}^\alpha _1(x)=\zeta \end{aligned}$$

and

6.13 $$\begin{aligned} \frac{C^{-1}}{(1+|x|)^{N+p}} \le \textsf{h}_1^\alpha (x) \le \frac{C}{(1+|x|)^{N+p}} \end{aligned}$$

for all $x\in \mathbb {R}^N$. Finally, we let ${\mathscr {C}}_p\subset {\mathscr {A}}$ be the subset of indices $\alpha \in {\mathscr {A}}$ such that there exist ${\bar{t}}_\alpha ,c_\alpha >0$, a Borel function $\psi _\alpha :(0,\infty )\rightarrow (0,\infty )$ and a measurable function $\kappa _\alpha :\mathbb {R}^N\rightarrow [0,\infty )$ such that

6.14 $$\begin{aligned} \frac{\textsf{h}_t^\alpha (x)}{\psi _\alpha (t)}\le c_\alpha \kappa _\alpha (x) \end{aligned}$$

for a.e. $x\in \mathbb {R}^N$ and $t\in (0,{\bar{t}}_\alpha )$ and

6.15 $$\begin{aligned} \lim _{t\rightarrow 0^+} \frac{\textsf{h}_t^\alpha (x)}{\psi _\alpha (t)} = \kappa _\alpha (x) \end{aligned}$$

for a.e. $x\in \mathbb {R}^N$. We also let $\widetilde{{\mathscr {C}}}_p\subset {\mathscr {C}}_p$ be the subset of indices $\alpha \in {\mathscr {C}}_p$ such that there exist ${\hat{t}}_\alpha ,{\hat{c}}_\alpha >0$ and a measurable function $\hat{\kappa }_\alpha :\mathbb {R}^N\rightarrow [0,\infty ]$ such that

6.16 $$\begin{aligned} \hat{\kappa }_\alpha \notin L^1(\mathbb {R}^N) \quad \text {and} \quad \hat{\kappa }_\alpha \in L^1(\mathbb {R}^N\setminus B_R) \end{aligned}$$

for all $R>0$, and

6.17 $$\begin{aligned} \frac{\textsf{h}_t^\alpha (x)}{\psi _\alpha (t)} \ge {\hat{c}}_\alpha \hat{\kappa }_\alpha (x) \end{aligned}$$

for a.e. $x\in \mathbb {R}^N$ and $t\in (0,{\hat{t}}_\alpha )$. As above, for each $\alpha \in {\mathscr {A}}$ and $p\in [1,\infty ]$, we define

$$\begin{aligned} (\textsf{H}_t^\alpha )_{t>0}:L^p(\mathbb {R}^N)\rightarrow L^p(\mathbb {R}^N) \end{aligned}$$

by letting

6.18 $$\begin{aligned} \textsf{H}_t^\alpha u=\textsf{h}_t^\alpha *u \end{aligned}$$

for all $u\in L^p(\mathbb {R}^N)$ and $t>0$. We observe that (6.18) makes sense by Young’s inequality, since $\textsf{h}_t^\alpha \in L^1(\mathbb {R}^N)$ for all $t>0$ and $\alpha \in {\mathscr {A}}$ by assumption.

We are now ready to state our result, improving and generalizing [2, Th. 1.1].

#### Theorem 6.2

Let $p\in [1,\infty )$ and let $(\textsf{H}_t^\alpha )_{t>0,\alpha \in {\mathscr {A}}}$ be as above. Let $\varsigma _\alpha :I\rightarrow [0,\infty )$ be defined as

$$\begin{aligned} \varsigma _\alpha (t) = {\left\{ \begin{array}{ll} t^{\beta p} &  \text {if}\ \alpha \in {\mathscr {A}}_p, \\ t^{\beta p}|\log t| &  \text {if}\ \alpha \in {\mathscr {B}}_p^{\zeta ,C}, \\ \psi _\alpha (t) &  \text {if}\ \alpha \in \widetilde{{\mathscr {C}}}_p, \end{array}\right. } \end{aligned}$$

for all $\alpha \in {\mathscr {A}}$, with $\zeta >0$ and $C\ge 1$. The limits

$$\begin{aligned} \lim _{t\rightarrow 0^+}\int _{\mathbb {R}^N} \frac{\textsf{H}_t^\alpha (|u-u(x)|^p)(x)}{\varsigma _\alpha (t)}\,\textrm{d}x = {\left\{ \begin{array}{ll} \displaystyle \frac{c_{N,p}}{N\,\Vert |\cdot |^p \,\textsf{h}_1^\alpha \Vert _{L^1}} \, \Vert Du\Vert _{L^p}^p &  \text {in}\ {\mathcal {S}^{p}}(\mathbb {R}^N)\ \text {if}\ \alpha \in {\mathscr {A}}_p, \\ \zeta \,\beta \,\omega _{N}\,c_{N,p} \, \Vert Du\Vert _{L^p}^p &  \text {in}\ {\mathcal {S}^{p}}(\mathbb {R}^N)\ \text {if}\ \alpha \in {\mathscr {B}}_p^{\zeta ,C}, \\ \displaystyle [u]_{W^{\kappa _\alpha ,p}}^p &  \text {in}\ W^{\kappa _\alpha ,p}(\mathbb {R}^N)\ \text {if}\ \alpha \in {\mathscr {C}}_p, \end{array}\right. } \end{aligned}$$

where

$$\begin{aligned} c_{N,p} = \frac{2\,\Gamma \left( \frac{p+1}{2}\right) \Gamma \left( \frac{N+1}{2}\right) }{\Gamma \left( \frac{N+p}{2}\right) }, \end{aligned}$$

hold in the pointwise sense and in the $\Gamma$-sense with respect to the $L^p$ topology, and all the functionals on the left-hand sides are coercive on the respective spaces. Moreover, if $(t_k)_{k\in \mathbb {N}}\subset I$ is infinitesimal and $(u_k)_{k\in \mathbb {N}}\subset L^p(\mathbb {R}^N)$ is such that

$$\begin{aligned} \liminf _{k\rightarrow \infty } \frac{1}{\varsigma _\alpha (t_k)} \int _{\mathbb {R}^N}\textsf{H}_{t_k}^\alpha (|u_k-u_k(x)|^p)(x)\,\textrm{d}x<\infty , \end{aligned}$$

then $(u_k)_{k\in \mathbb {N}}$ is locally precompact in $L^p(\mathbb {R}^N)$ and any of its $L^p_\textrm{loc}(\mathbb {R}^N)$ limits is in ${\mathcal {S}^{p}}(\mathbb {R}^N)$ if $\alpha \in {\mathscr {A}}_p\cup {\mathscr {B}}_p^{\zeta ,C}$ and in $W^{\kappa _\alpha ,p}(\mathbb {R}^N)$ if $\alpha \in \widetilde{{\mathscr {C}}_p}$.

#### Proof

The proof is quite similar to that of Theorem 1.7, so we simply sketch it. As before, letting $K^\alpha (x)=\textsf{h}_1^\alpha (x)$ for all $x\in \mathbb {R}^N$ and $\beta _\alpha (t)=t^{-\beta }$ for all $t>0$, we get that $K_t^\alpha (x)=\textsf{h}^\alpha _t(x)$ for all $x\in \mathbb {R}^N$ and $t>0$. The case $\alpha \in {\mathscr {A}}_p$ directly follows from Theorems 4.2 and 1.3. For the case $\alpha \in {\mathscr {B}}_p^{\zeta ,C}$, we observe that, owing to (6.12),

$$\begin{aligned} \phi _{K^\alpha ,p}(t) \sim N\omega _N\,\zeta \beta \,t^{\beta p}|\log t| \quad \text {as}\ t\rightarrow 0^+. \end{aligned}$$

Therefore, it is enough to check that the family $(\rho _t)_{t>0}$ given by

$$\begin{aligned} \rho _t(x) = \frac{|x|^p\,\textsf{h}_t^\alpha (x)}{t^{\beta p}|\log t|}, \quad \text {for all}\ x\in \mathbb {R}^N\ \text {and}\ t>0, \end{aligned}$$

satisfies the properties in Theorem 1.1(A) (with no need of passing to a subsequence). For the compactness part, thanks to (6.13), it is enough to apply Lemma 4.6. Finally, owing to (6.14) and (6.15), the case $\alpha \in {\mathscr {C}}_p$ follows from Lemma 1.4, while, owing also to (6.16) and (6.17), the compactness in the case $\alpha \in \widetilde{{\mathscr {C}}}_p$ follows from [17, Th. 2.11]. $\square$

#### Remark 6.3

(*Comparison with* [2, Th. 1.1]) We observe that [2, Th. 1.1] deals with the case $p=1$ only, and only in the case of bounded sets of finite perimeter. In addition, [2, Th. 1.1(ii)] is weaker than Lemma 6.2, as [2, Th. 1.1(ii)] provides an upper estimate on the $\limsup$ and only under the stronger assumption that

6.19 $$\begin{aligned} \textsf{h}_1^\alpha (x)=\frac{c}{(1+|x|^n)^m}, \quad \text {for all } x\in \mathbb {R}^N \end{aligned}$$

for some $c,n,m>0$ such that $mn=N+1$. Our result instead relies on (6.12) and (6.13) only, which, in turn, in the case (6.19), naturally impose that $mn=N+p$. We further remark that our renormalization of $(\textsf{h}_t^\alpha )_{\alpha \in {\mathscr {A}},t>0}$ differs from the one adopted in [2], as we require that $\Vert \textsf{h}_1^\alpha \Vert _{L^1}=1$ for all $\alpha \in {\mathscr {A}}$. However, due to the scaling assumption (6.11), corresponding to [2, Eq. (1.1)], the two approaches are completely equivalent.

## Heat Content in Hilbert Spaces

In this last section we briefly provide a different point of view on the sufficient condition achieved in Theorem 1.1 in the Hilbertian framework.

### Heat Content in Hilbert Spaces

We recall some standard notation (for a more detailed account, see, e.g., [40]). We let ${\mathcal {H}}$ be a Hilbert space with scalar product $(\,\cdot ,\,\cdot \,)_{{\mathcal {H}}}$ and we let $(\textsf{H}_t)_{t\ge 0}$ be a *strongly continuous semigroup of symmetric operators* on ${\mathcal {H}}$.

#### Definition 7.1

(*Semigroup content*) The *semigroup content* of $u\in {\mathcal {H}}$ is the map $[0,\infty )\ni t\rightarrow {{\mathbb {H}}}_t(u)\in [0,\infty )$ defined as

$$\begin{aligned} {{\mathbb {H}}}_t(u) = (\textsf{H}_t u,u)_{{\mathcal {H}}} \end{aligned}$$

for all $t\ge 0$. In particular, $0\le {{\mathbb {H}}}_t(u)\le {{\mathbb {H}}}_0(u)= (u,u)_{{\mathcal {H}}}$ for $t\ge 0$ and $u\in {\mathcal {H}}$.

We let $L$ be the *generator* of the semigroup $(\textsf{H}_t)_{t\ge 0}$, which is given by

7.1 $$\begin{aligned} Lu=\lim _{t\rightarrow 0^+}\frac{\textsf{H}_tu-u}{t} \quad \text {in}\ {\mathcal {H}}, \end{aligned}$$

with domain ${\mathcal {D}}(L)=\left\{ u\in {\mathcal {H}}: Lu~\text {exists as a strong limit}\right\}$. We recall that $L$ is a non-positive definite self-adjoint operator, see [40, Lem. 1.3.1]. The notions of convergence in ${\mathcal {D}}(L)\subset {\mathcal {H}}$ in the pointwise and in the $\Gamma$-sense with respect to the topology in ${\mathcal {H}}$ are the natural analogues of Definitions 2.3 and 2.4. We omit the plain statements.

With this notation in force, we can prove Theorem 1.8.

#### Proof of Theorem 1.8

Let $E_\cdot =(E_\lambda )_{\lambda \ge 0}$ be the *spectral representation* of $-L$, so that

$$\begin{aligned} (-Lu,v)_{{\mathcal {H}}}=\int _0^\infty \lambda \,\textrm{d}(E_\lambda u,v)_{{\mathcal {H}}} \end{aligned}$$

for $u\in {\mathcal {D}}(L)$ and $v\in {\mathcal {H}}$. By [40, Lem. 1.3.2], we can write

$$\begin{aligned} (\textsf{H}_t u,v)_{{\mathcal {H}}} = \int _0^\infty e^{-t\lambda }\,\textrm{d}(E_\lambda u,v)_{{\mathcal {H}}}, \end{aligned}$$

for all $t\ge 0$ and $u,v\in {\mathcal {H}}$, and thus

$$\begin{aligned} {{\mathbb {H}}}_0(u)-{{\mathbb {H}}}_t(u) = (u-\textsf{H}_t u,u)_{{\mathcal {H}}} = \int _0^\infty (1-e^{-t\lambda })\,\textrm{d}(E_\lambda u,u) \end{aligned}$$

for all $t\ge 0$ and $u\in {\mathcal {H}}$. Since $1-s\le e^{-s}$ for all $s\ge 0$, we can hence estimate

$$\begin{aligned} {{\mathbb {H}}}_0(u)-{{\mathbb {H}}}_t(u) \le t \int _0^\infty \lambda \,\textrm{d}(E_\lambda u,u) = t (-Lu,u)_{{\mathcal {H}}} \end{aligned}$$

for all $t\ge 0$ and $u\in {\mathcal {D}}(L)$, yielding (i).

Now let $(u_k)_{k\in \mathbb {N}}\subset {\mathcal {H}}$, $u\in {\mathcal {H}}$ and $(t_k)_{k\in \mathbb {N}}\subset (0,\infty )$ be as in (ii). We define the measures $\mu _k,\mu \in {\mathscr {M}}_\textrm{loc}^+((0,\infty ))$ by letting $\mu _k=(E_{\cdot }u_k,u_k)_{{\mathcal {H}}}$ for all $k\in \mathbb {N}$ and $\mu =(E_{\cdot }u,u)_{{\mathcal {H}}}$. Since $u_k\rightarrow u$ in ${\mathcal {H}}$ as $k\rightarrow \infty$, we infer that $\mu _k\overset{\star }{\rightharpoonup }\mu$ in ${\mathscr {M}}_\textrm{loc}((0,\infty ))$ as $k\rightarrow \infty$. Therefore, given $R>0$, we can estimate

$$\begin{aligned} \begin{aligned} \frac{{{\mathbb {H}}}_0(u_k)-{{\mathbb {H}}}_{t_k}(u_k)}{t_k} \ge \int _0^R \frac{1-e^{-t_k\lambda }}{t_k\lambda }\,\lambda \,\textrm{d}\mu _k(\lambda ) \ge \frac{1-e^{-t_kR}}{t_kR} \int _0^R \lambda \,\textrm{d}\mu _k(\lambda ) \end{aligned} \end{aligned}$$

for all $k\in \mathbb {N}$. By Tonelli’s Theorem and Fatou’s Lemma, we have that

$$\begin{aligned} \liminf _{k\rightarrow \infty } \int _0^R \lambda \,\textrm{d}\mu _k(\lambda ) = \liminf _{k\rightarrow \infty } \int _0^R\mu _k((t,R))\,\textrm{d}t \ge \int _0^R\mu ((t,R))\,\textrm{d}t = \int _0^R\lambda \,\textrm{d}\mu (\lambda ) \end{aligned}$$

for all $R>0$. As a consequence, we get that

$$\begin{aligned} \liminf _{k\rightarrow \infty } \frac{{{\mathbb {H}}}_0(u_k)-{{\mathbb {H}}}_{t_k}(u_k)}{t_k} \ge \int _0^R\lambda \,\textrm{d}\mu (\lambda ) \end{aligned}$$

for all $R>0$. Letting $R\rightarrow \infty$ in the above inequality, we conclude that

$$\begin{aligned} \liminf _{k\rightarrow \infty } \frac{{{\mathbb {H}}}_0(u_k)-{{\mathbb {H}}}_{t_k}(u_k)}{t_k} \ge \int _0^\infty \lambda \,\textrm{d}\mu (\lambda ) = (-Lu,u)_{{\mathcal {H}}}, \end{aligned}$$

proving (ii). The rest of the statement follows by choosing $u_k=u$ for all $k\in \mathbb {N}$. $\square$

### A Quicker Approach via Fourier Transform

We now provide an alternative quicker proof of Theorem 1.8 for ${\mathcal {H}}=L^2(\mathbb {R}^N)$ via the Fourier transform.

We let

$$\begin{aligned} {\mathcal {F}}(u)(\xi ) = {\hat{u}}(\xi ) =\int _{\mathbb {R}^N}e^{-2\pi ix\cdot \xi }\,u(x)\,\textrm{d}x, \end{aligned}$$

for all $\xi \in \mathbb {R}^N$, be the *Fourier transform* of $u\in L^1(\mathbb {R}^n)$. As customary, we extend the Fourier transform to a unitary transformation on $L^2(\mathbb {R}^N)$ keeping the same notation.

Given a measurable function $\lambda :\mathbb {R}^N\rightarrow [0,\infty ]$, the family

$$\begin{aligned} (\textsf{H}_t)_{t\ge 0}:L^2(\mathbb {R}^N)\rightarrow L^2(\mathbb {R}^N), \end{aligned}$$

defined as

7.2 $$\begin{aligned} (\textsf{H}_t u,v)_{L^2} = \int _{\mathbb {R}^N}e^{-\lambda (\xi )t}\,{\hat{u}}(\xi )\cdot \overline{{\hat{v}}(\xi )}\,\textrm{d}\xi \end{aligned}$$

for all $t\ge 0$ and $u,v\in L^2(\mathbb {R}^N)$, yields a strongly continuous semigroup of symmetric operators on $L^2(\mathbb {R}^N)$. As in Definition 7.1, we can thus define the semigroup content corresponding to (7.2) as

$$\begin{aligned} {{\mathbb {H}}}_t(u) = (\textsf{H}_tu,u)_{L^2} = \int _{\mathbb {R}^N}e^{-\lambda (\xi )t}\,|{\hat{u}}(\xi )|^2\,\textrm{d}\xi \end{aligned}$$

for all $t\ge 0$ and $u\in L^2(\mathbb {R}^N)$. Moreover, as in (7.1), the generator of (7.2) is given by

$$\begin{aligned} (-Lu,v)_{L^2} = \int _{\mathbb {R}^N}\lambda (\xi )\,{\hat{u}}(\xi )\cdot \overline{{\hat{v}}(\xi )}\,\textrm{d}\xi , \quad v\in L^2(\mathbb {R}^N), \end{aligned}$$

for all $u\in {\mathcal {D}}(L)$, where ${\mathcal {D}}(L)=\left\{ u\in L^2(\mathbb {R}^N): \lambda |{\hat{u}}|\in L^2(\mathbb {R}^N)\right\}$. As usual, we can interpret $\lambda$ as the *Fourier symbol* of the (non-negative) operator $-L$.

#### Alternative proof of Theorem 1.8

 Since we have

$$\begin{aligned} {{\mathbb {H}}}_0(u)-{{\mathbb {H}}}_t(u) = \int _{\mathbb {R}^N}(1-e^{-\lambda (\xi )t})|{\hat{u}}(\xi )|^2\,\textrm{d}\xi \end{aligned}$$

for all $t\ge 0$ and $u\in L^2(\mathbb {R}^N)$, by the Dominated Convergence Theorem we infer that

$$\begin{aligned} \lim _{t\rightarrow 0^+} \frac{{{\mathbb {H}}}_0(u)-{{\mathbb {H}}}_t(u)}{t} = \int _{\mathbb {R}^N}\lambda (\xi )\,|{\hat{u}}(\xi )|^2\,\textrm{d}\xi = (-Lu,u)_{L^2} \end{aligned}$$

for all $u\in {\mathcal {D}}(L)$. Now let $(t_k)_{k\in \mathbb {N}}\subset (0,\infty )$ be infinitesimal and $(u_k)_{k\in \mathbb {N}}\subset L^2(\mathbb {R}^N)$ be such that $u_k\rightarrow u$ in $L^2(\mathbb {R}^N)$ as $k\rightarrow \infty$ with $u\in {\mathcal {D}}(L)$. As a consequence, also ${\hat{u}}_k\rightarrow {\hat{u}}$ in $L^2(\mathbb {R}^N)$ as $k\rightarrow \infty$ and thus, owing to Plancherel’s Theorem, up to passing to a subsequence, ${\hat{u}}_k(\xi )\rightarrow {\hat{u}}(\xi )$ for a.e. $\xi \in \mathbb {R}^N$ as $k\rightarrow \infty$. Then, by Fatou’s Lemma, we get

$$\begin{aligned} \liminf _{k\rightarrow \infty }\frac{{{\mathbb {H}}}_0(u_k)-{{\mathbb {H}}}_{t_k}(u_k)}{t_k}= &   \liminf _{k\rightarrow \infty } \int _{\mathbb {R}^N}\frac{1-e^{-\lambda (\xi )t_k}}{t_k}\,|{\hat{u}}_k(\xi )|^2\,\textrm{d}\xi \\\ge &   \int _{\mathbb {R}^N}\lambda (\xi )|{\hat{u}}(\xi )|^2\,\textrm{d}\xi , \end{aligned}$$

readily yielding the conclusion. $\square$

#### Remark 7.2

(*Application to heat kernels*) The choice $\lambda (\xi )=4\pi ^2|\xi |^2$ for all $\xi \in \mathbb {R}^N$ yields that $\textsf{H}_tu=\textsf{h}_t*u$ for all $t\ge 0$ and $u\in L^2(\mathbb {R}^N)$ as in (6.2), and $L=\Delta$ is the Laplacian operator. Similarly, given $s\in (0,1)$, the choice $\lambda (\xi )=(2\pi |\xi |)^{2s}$ for all $\xi \in \mathbb {R}^N$, yields that $\textsf{H}_t^su=\textsf{h}_t^s*u$ for all $t\ge 0$ and $u\in L^2(\mathbb {R}^N)$ as in (6.10), and $L=-(-\Delta )^{s}$ is the *fractional Laplacian operator*, see [68] for instance.

#### Remark 7.3

(*Characteristic functions*) In the setting of Sect. 7.2, Theorem 1.8 can be applied to characteristic functions of sets with finite volume. In particular, in the case of heat kernels as in Remark 7.2, this point of view allows to recover the results of [47] for $s\in \left( 0,\frac{1}{2}\right)$. For finer results in this direction, we also refer to [16].

#### Remark 7.4

(*Non-negativity assumption*) It is worth observing that the non- negativity assumption $-L\ge 0$ in Sect. 7.1 (or, analogously, the fact that $\lambda \ge 0$ in (7.2) in Sect. 7.2) plays a crucial role in the proof of Theorem 1.8. We do not know if such non-negativity assumption can be dropped or, at least, relaxed. Similar considerations can be made concerning the non-negativity assumption $\rho _t\ge 0$ for $t\in I$ in the case of the functionals $({\mathscr {F}}_{t,p})_{t\in I}$ in (2.8) for $p\in [1,\infty )$. We refer to [4, 5] for related discussions. The authors thank Giovanni Alberti for several observations about these aspects.

## Data Availability

No new data were created or analysed in this study. Data sharing is not applicable to this article.
